# Working Hydration as a Design Variable in Hydrogel Fibers: Coupled Transport, Signal Fidelity, and Wearable Electronics

**DOI:** 10.3390/gels12100863

**Published:** 2026-09-24

**Authors:** Yaqin Tian, Wanxue Sun, Liyin Hou, Shanshan Guo, Xiaojuan Wang, Caixia Ren, Zhe Cui

**Affiliations:** 1 Shandong Engineering Research Center of Low-Carbon Energy Internet of Things Technology & School of Energy and Constructional Engineering, Shandong Huayu University of Technology, Dezhou 253034, China; 2 Shandong Key Laboratory of Intelligent Manufacturing Technology for Advanced Power Equipment, Weifang University, Weifang 261061, China; 3 Christopher Ingold Laboratory, Department of Chemistry, University College London, London WC1H 0AJ, UK

**Keywords:** hydrogel fibers, hydration, coupled transport, iontronics, wearable electronics, signal fidelity, textile integration, reliability

## Abstract

Hydrogel fibers combine a hydrated medium resembling tissue with axial continuity, anisotropy, and textile processability. Yet performance is often evaluated at a nominal water content that cannot represent water redistribution during operation. Unlike previous reviews centered on composition, fabrication, or application, this Review uses the working hydration state to link network evolution, transport, signal generation, integration, and reliability. It refers to water distribution, physicochemical state, and exchange dynamics under operating conditions. Four themes are examined: network evolution induced by hydration, axial and radial transport, signal generation and decoupling, and preservation of function from fibers to textiles and biointerfaces. Comparative analysis indicates that reliable operation requires preserved axial pathways, controlled radial exchange, spatial separation of coupled signals, reference channels, and calibration after hydration and deformation histories. Direct fiber evidence is distinguished from transferable evidence from soft devices. Major gaps include inconsistent equilibration criteria, nonstandard hydration cycling, insufficient synchronized operando measurements, limited interlaboratory reproducibility and human validation over extended periods, and scarce manufacturing statistics. Future research should prioritize models that account for hydration, standardized service testing, scalable textile processing, and validation across devices and users. This framework establishes working hydration as a basis for material design, signal interpretation, and reliability assessment.

## 1. Introduction

Wearable and flexible electronics must operate as durable biointerfaces rather than merely survive bending or stretching. Mechanical conformity, electrical coupling, and chemical exchange must persist while skin or tissue moves, sweats, and experiences changes in temperature and humidity. Hydrogels reduce mechanical mismatch with biological surfaces and provide hydrated pathways for ionic and molecular communication [1,2]. All-hydrogel microfluidics and wet-adhesive interfaces further support fluid handling and conformal attachment [3,4]. The unresolved problem is that dehydration, uncontrolled swelling, freezing, sweat contamination, and repeated deformation can change network dimensions, transport, adhesion, and contact impedance at the same time. These coupled changes can produce signal drift, hysteresis, environmental cross-sensitivity, and device-to-device variation even when the fiber remains macroscopically intact. Consequently, low initial modulus, high conductivity, or strong adhesion does not establish reliable function after fabrication, attachment, repeated deformation, perspiration, environmental exposure, and prolonged wear [5,6]. The central materials problem is thus the preservation of an identifiable and calibrated signal pathway throughout the operating lifetime.

Hydrogel fibers address this problem through a geometry that couples long-range axial continuity with short-range radial exchange [7]. Spinning, drawing, extrusion, and post-treatment can align polymer chains, pores, crystalline domains, and conductive components along the fiber axis, supporting the transmission of stress, charge, or light over macroscopic distances. The small radius shortens the diffusion path for water, ions, metabolites, and other molecular species, which can accelerate local sampling from the surrounding medium. Fibers can also be twisted, knotted, woven, knitted, or braided to distribute sensing, transmission, actuation, and mechanical support within deformable fabrics. The same geometry creates a fundamental trade-off. A large surface-area-to-volume ratio promotes rapid exchange but also increases susceptibility to dehydration, uncontrolled swelling, contamination, and medium-dependent changes in conductivity and mechanics. The functional advantage of the fiber depends on controlling radial exchange without sacrificing axial pathway continuity.

Research has progressed from establishing spinnability to controlling network organization, transport pathways, soft biointerfaces, textile integration, and hydration-dependent operation. Persistent and dynamic interactions can separately support load bearing, energy dissipation, and conductive-pathway retention [8,9]. Studies of soft biointerfaces show that recording quality depends on time-dependent contact area, interfacial stress, hydration, and ionic impedance [10,11], while patterned all-hydrogel conductors define soft, addressable transport pathways within integrated devices [12]. Representative reviews have summarized conductive hydrogel fibers, wearable sensors and soft actuators, polymer-specific conducting hydrogels, and temperature-sensing platforms [13,14,15,16]. A recent PVA/cellulose-nanofibril hydrogel further demonstrates how hydration-sensitive hydrogen-bonded junctions couple mechanical deformation to electrical output [17]. Collectively, these studies show that reliability is governed across multiple scales, from molecular control of solvent and charge mobility to fiber-level transport, textile-level deformation and solvent exchange, and biointerface-level contact mechanics and ionic coupling. However, previous reviews remain primarily organized around material classes, fabrication routes, conductive chemistries, or applications and do not integrate these scales through a common operating-state variable. The distinctive contribution of the present Review is a causal framework that follows the working hydration state from water redistribution through network evolution, axial and radial transport, signal generation, integration, and retained calibration. Rather than ranking initial performance, we ask whether the same signal pathway remains interpretable after defined hydration, deformation, and environmental histories. Table 1 compares these representative reviews by scope, primary organization, treatment of hydration, and contribution relative to the present Review.

The hydrated state responsible for softness and ionic functionality is not a fixed material property. Water acts as a plasticizer, ionic solvent, analyte carrier, optical medium, and regulator of polymer–polymer and polymer–filler interactions. Its redistribution can change fiber diameter, porosity, modulus, ionic mobility, electronic percolation, interfacial impedance, and optical response within the same measurement. Some of these changes are functional. Analyte-induced swelling or ion exchange may generate the desired response. Others are parasitic because evaporation, perspiration, humidity variation, or unintended fluid uptake can alter the same transport and structural variables, producing baseline drift, hysteresis, crosstalk, or false signals. Initial conductivity, elongation, gauge factor, response time, and skin-contact impedance cannot distinguish functional from parasitic changes when measured on different specimens or under narrowly controlled conditions. We use working hydration state to denote the spatial distribution, physicochemical state, and exchange dynamics of water and solvated species under the actual mechanical, electrical, thermal, and chemical boundary conditions of operation. To avoid overlapping reliability terminology, signal fidelity denotes preservation of the magnitude, timing, and identity of the target response after nontarget disturbances; signal stability describes time dependence under a constant input and specified boundary conditions; reversibility requires recovery after removal of a perturbation; survivability requires only continued function after exposure; and reliability denotes retention of the required fidelity over the declared wear period and operating envelope.

This Review treats working hydration as the organizing variable for hydrogel-fiber bioelectronics. Figure 1 maps the causal sequence linking composition and network architecture to solvent distribution, axial and radial transport, transduction, assembly, interfaces, and reliability. Section 2 and Section 3 establish how processing and cross-sectional design create the hydrated structure and its transport pathways. Section 4 examines signal generation, drift, hysteresis, crosstalk, and decoupling, while Section 5 and Section 6 address textile integration and service reliability. Section 7 evaluates applications according to the pathway supported by the reported evidence and the validation boundary that remains. Nonfibrous hydrogels are considered only when their mechanisms are transferable to fibers, yarns, textiles, or their interfaces. This boundary keeps the discussion focused on how hydration-dependent structural evolution governs signal generation, interpretation, and retention.

This article is a narrative, mechanism-oriented Review rather than a systematic review or meta-analysis. The literature corpus was assembled through searches of Web of Science Core Collection, Scopus, PubMed, and Google Scholar using combinations of hydrogel fiber, wet spinning, ionic or electronic conduction, hydration, water retention, swelling, biointerface, wearable sensor, smart textile, signal drift, and reliability. Studies published mainly from January 2018 through August 2026 were considered, with earlier foundational reports retained when they established a mechanism or benchmark used by later work. Priority was given to studies that reported composition and geometry together with test conditions, quantitative transport or mechanical data, and a hydration-dependent outcome. Direct fiber evidence is distinguished from transferable evidence obtained from bulk hydrogels, patches, and related soft devices, and the latter is not used to claim direct fiber validation. Mechanistic statements are phrased according to evidence strength as demonstrated, consistent with, or proposed. Because protocols and reporting units remain heterogeneous, the synthesis is comparative and mechanistic rather than statistically pooled.

## 2. Hydration and Structural Evolution in One-Dimensional Networks

Hydrogel fibers are often classified by polymer composition, crosslinking chemistry, or fabrication route. These categories remain useful for synthesis, but they do not define the physical state in which a fiber generates and transmits a signal. Spinning, washing, storage, integration, and wear alter solvent content, polymer organization, conductive contacts, and interfacial boundary conditions. Fibers with similar nominal compositions may consequently operate in different mechanical and transport regimes. This section examines how water populations, one-dimensional geometry, processing-induced anisotropy, and post-fabrication network evolution establish the working structure that governs subsequent transport and transduction.

### 2.1. Water States and Polymer Hydration

Water in a hydrogel does not constitute a single thermodynamic population. Relatively mobile water occupies open pores and supports rapid molecular diffusion, whereas weakly associated water undergoes transient exchange with hydrophilic polymer groups. More strongly bound water forms persistent hydration shells or confined hydrogen-bonded domains, while solvating water coordinates mobile ions and influences their local dissociation and transport. The proportions and exchange rates of these water populations determine whether a hydrogel fiber favors rapid transport, solvent retention, or a balance between the two. A larger fraction of mobile water generally promotes ionic mobility and analyte exchange, but it also increases susceptibility to evaporation and composition drift. Stronger polymer–water interactions can suppress freezing and preserve flexibility below 0 °C, although excessive association may increase the activation barrier for ion migration. Equilibrium water content alone is insufficient to define the operating state of a fiber. Hydration measurements should be accompanied by the test temperature, relative humidity, equilibration time, solvent composition, and preceding sorption or drying history. Equilibration time is especially important for fibers because the short radial diffusion distance can create the appearance of rapid stabilization while a coating, compact yarn, or asymmetric skin/sweat boundary maintains axial and cross-sectional water gradients. A reported property should be assigned either to a defined transient time after exposure or to an equilibrium criterion, such as a specified rate of mass or dimensional change. Without that distinction, conductivity or sensitivity measured at different conditioning times may represent different operating structures rather than reproducible material differences.

Network chemistry regulates these water populations through polymer hydrophilicity, fixed-charge density, hydrogen bonding, reversible coordination, and the amount and distribution of crystalline or semicrystalline domains. Ionic and dynamic covalent junctions allow local bond exchange and energy dissipation, whereas crystallites, aligned nanofibers, and other persistent domains provide a load-bearing framework that is less sensitive to short-term solvent fluctuations. Interpenetrating and hierarchical networks have consequently emerged as an important design direction because mechanical support and charge transport can be assigned to different but mechanically coupled phases [13,14]. This separation is particularly relevant to hydrogel fibers. Radial solvent exchange can continuously alter the spacing and connectivity of conductive domains, while axial deformation simultaneously challenges the continuity of the load-bearing and signal-transmission pathways.

The relationship between solvent state and the intended electrical readout is illustrated by two contrasting fiber sensors. In a cryogenic artificial-nerve fiber, a dynamic ionic network combined with a low-freezing solvent phase retained chain mobility and ionic transport at subzero temperatures. The network could consequently restore both mechanical and electrical continuity after damage under cryogenic conditions [18]. This result shows that low-temperature sensing cannot be explained by room-temperature conductivity alone. The solvent must remain sufficiently mobile to sustain ion migration and permit reversible network reconstruction. A pressure/proximity bimodal fiber addressed a different aspect of signal reliability by separating contact-induced deformation from non-contact electric-field perturbation. Its proximity channel reached a sensitivity of 3.17% cm^−1^ over a sensing distance of 18 cm, with response and recovery times of approximately 90 ms [19]. Although this study did not explicitly quantify individual water populations, it highlights a related device-level requirement. Preserving conductivity is not sufficient if changes in the hydrated network cause responses to different stimuli to become indistinguishable.

Hydration also alters the mechanical relaxation spectrum of the fiber. Additional water commonly lowers local relaxation barriers, increases segmental mobility, and decreases the effective modulus. This relationship may become nonmonotonic when swelling activates new intermolecular associations, redistributes reversible junctions, or allows crystallites to reorganize. Dehydration can increase apparent stiffness and tensile strength while reducing extensibility, fracture tolerance, and interfacial conformity. A fiber that performs well at one relative humidity may become brittle after drying or excessively compliant after sweat uptake. These changes can shift the strain distribution, contact area, conductive-pathway spacing, and interfacial impedance even when the fiber remains macroscopically intact. Sorption and desorption hysteresis, equilibration time, diameter recovery, stress relaxation, and spatial water gradients should be measured together to determine whether a change in mechanical response reflects a reversible hydration state or accumulated structural damage.

### 2.2. Dimensional and Interfacial Effects

In fibers, hydration-dependent behavior is strongly influenced by one-dimensional geometry. Water, ions, and analytes generally exchange across the relatively short radial direction, whereas mechanical loading and signal transmission occur primarily along the fiber axis. Under an ideal diffusion-controlled approximation, the characteristic equilibration time follows *t*_d_∼*L*^2^/*D*_eff_, where L is the operative transport distance and *D*_eff_ is the effective diffusivity within the swollen network. Halving the fiber radius would reduce the ideal diffusion time to approximately one quarter, provided that transport across the external surface is not rate-limiting. Actual equilibration can differ substantially from this estimate because surface resistance, pore tortuosity, polymer–solute interactions, local confinement, and swelling-induced changes in diffusivity alter the operative transport pathway. This radial advantage may also diminish after device assembly. Skin contact, surface coatings, compact yarn packing, encapsulation, and textile junctions reduce the exposed surface area and produce nonuniform hydration boundary conditions. For example, skin may suppress evaporation from one side of a fiber while sweat enters from another, and the interior of a densely packed yarn may equilibrate more slowly than its surface even when each filament has a small diameter. Measurements on isolated fibers should be complemented by tests on yarns, textiles, and complete devices under the temperature, humidity, mechanical constraint, and exposure conditions expected during use.

Cross-sectional architecture determines how the short radial transport distance is used and which hydration-dependent failure mode is suppressed. Open pores increase accessible area and shorten local diffusion paths, but they can accelerate evaporation and weaken the swollen network. Hollow fibers introduce an axial channel for liquid delivery or storage within a radially permeable wall. Core–shell structures can combine a conductive hydrated core with a water-retaining, reinforcing, or selectively permeable outer layer, while Janus and gradient fibers create asymmetric solvent exchange, directional swelling, or preferential tissue contact. Outer diameter alone is an inadequate comparison variable. The operative transport distance and hydration response also depend on wall thickness, pore connectivity, shell permeability, interfacial bonding, exposed surface fraction, and the dimensions of each functional region. Performance should be assessed by locating where solvent exchange, charge transport, deformation, and interfacial impedance are controlled within the cross-section.

Internal pathway stabilization is illustrated by the aramid nanofiber (ANF)/poly(vinyl alcohol) (PVA) framework in Figure 2a,b, which templated a continuous polypyrrole (PPy) network and produced electrical percolation near 1 wt% PPy [20]. At approximately 65 wt% water, the optimized hydrogel reached an electronic conductivity of about 8000 S m^−1^, a tensile strength of 9.4 MPa, and an elongation at break of 36%. Its conductivity exceeded that of PPy hydrogels prepared without the ANF template by two to three orders of magnitude under comparable polymerization conditions [20], linking transport to spatial connectivity rather than dense occupation of the hydrated pore volume. The electrode also showed an area-normalized impedance of approximately 20 Ω cm^2^ at 50 Hz and a charge-injection capacity of 1.2 mC cm^−2^, compared with 93 Ω cm^2^ and 0.1 mC cm^−2^ for a thin gold electrode. These measurements distinguish bulk electronic continuity from tissue-facing charge transfer, two properties that may evolve differently during drying or swelling.

The external boundary requires a different control variable. A cellulose-nanofiber/dopamine/poly(acrylic acid)@Fe^3+^ hydrogel retained 80 wt% water and an ionic conductivity of 1.2 S m^−1^, while reversible Fe^3+^ coordination supplied cohesion and dopamine promoted adhesion (Figure 2c) [21]. Ultraviolet exposure reduced interfacial toughness from 89.13 to 5.82 J m^−2^, shear strength from 119.42 to 6.91 kPa, and tensile adhesion strength from 74.79 to 13.10 kPa. Adhesion recovered after approximately 5 min of air oxidation and remained switchable for five irradiation–oxidation cycles. The evidence supports reversible attachment without removing the hydrated ionic phase, although five cycles are insufficient to establish long-term interfacial durability. Integration with four triboelectric units, which charged a 0.1 mF capacitor to 3 V in approximately 5 h before wireless motion classification, further shows that hydration and adhesion control can survive system assembly while also exposing the limited autonomous duty cycle [21].

The two systems support a spatially differentiated design rule rather than a direct performance comparison. Internal conductive continuity, solvent retention, mechanical reinforcement, electrochemical transfer, and tissue adhesion need not be supplied by one homogeneous network. A protected internal domain can carry load or charge, while an external domain controls water exchange, impedance, and attachment. Hollow and core–shell fibers implement this separation through radial compartmentalization [22,23], and Janus or gradient fibers add directional exchange and swelling. The relevant assessment is spatial and time-dependent. Local hydration, diameter evolution, axial conductivity, radial diffusivity, interfacial impedance, and strain distribution should be measured under the same boundary conditions to determine whether compartmentalization stabilizes the intended pathway or merely delays equilibration.

### 2.3. Spinning-Induced Anisotropy and Radial Heterogeneity

Spinning establishes molecular and mesoscale organization rather than merely defining external shape. Shear and extensional flow orient polymer chains, nanofibers, and conductive fillers along the fiber axis, while coagulation and solvent exchange reorganize the network across the radius. Rapid precipitation may form a dense skin around a more hydrated core; inward diffusion of crosslinkers can generate a radial junction gradient; post-drawing can align crystallites and reversible associations. These structures influence anisotropic swelling, stress relaxation, fracture, and electrical stability after fabrication. Processing should consequently be reported with final diameter, draw ratio, dope composition, coagulation-bath chemistry, solvent-exchange history, post-treatment, and working hydration state. Axial conductivity measured without the corresponding diameter change, swelling ratio, and cyclic resistance drift cannot establish whether processing-induced alignment survives radial solvent exchange.

The sodium-polyacrylate system in Figure 3a–f shows how solvent exchange and chain conformation can program mechanics without changing the base polymer [24]. Dopes containing 3.5 wt% sodium polyacrylate at pH 3.95–13.97 were extruded into methanol, and water left the nascent fibers within approximately 150–250 s. Subsequent moisture uptake produced equilibrium water contents of about 12–40 wt%. Across these states, elongation ranged from 105 ± 2% to 2630 ± 120%, strength from 1.21 ± 0.12 to 47 ± 5 MPa, modulus from 0.240 ± 0.030 to 2050 ± 370 MPa, and toughness from 1.7 ± 1.1 to 17.8 ± 1.6 MJ m^−3^. At pH 13.34, the breaking strain and toughness reached 2693% and 20.3 MJ m^−3^, respectively. The range is consistent with transitions among aggregated coils, interpenetrated chains, and aligned domains. Coupling regions with mismatched deformation converted this molecular programming into helical uncoiling, but comparisons among pH states remain conditional on their different equilibrium water contents.

Radial compartmentalization preserves processing-induced organization by slowing solvent loss at the fiber boundary. A 4 wt% sodium-polyacrylate solution containing 20 wt% dimethyl sulfoxide formed a 200 ± 20 μm hydrated core with a 2–3 μm poly(methyl acrylate) shell [25]. At 25 °C and 42 ± 2% relative humidity (RH), with 25 ± 3 wt% retained water, the fiber exhibited a tensile strength of 5.6 ± 0.6 MPa, an elongation at break of 1180 ± 100%, and a toughness of 26.8 ± 3.1 MJ m^−3^. The stress–strain curve nearly recovered within 30 s after loading to 300% strain, while ionic conductivity increased from approximately 2 S m^−1^ to almost four times that value at 300% strain. Diffraction peaks at 2θ = 6.4°, 13.7°, and 17.0°, together with a fivefold increase in the relative intensity of the 13.7° peak during stretching, are consistent with strain-induced ordering. Persistent ordered regions and mobile amorphous chains can support continuity and reversible alignment, but the shell’s transport penalty still requires time-resolved sorption and response measurements.

These examples define two distinct stabilization routes. Conformational and helical programming redistributes axial strain, whereas a radial barrier moderates solvent exchange. Reinforcing fibers, conductive nanofibrillar scaffolds, and functional coatings preserve electrical continuity through related load-transfer mechanisms [23,26,27]. Multiply crosslinked cellulose/poly(vinyl alcohol) networks and MXene organohydrogels instead regulate swelling, ion motion, and low-temperature operation [28,29,30]. Textile studies add a further constraint because alignment must survive yarn formation, bending, friction, and repeated contact at knitted junctions [31,32]. Energy-storage, energy-harvesting, and optical fibers assign mechanical support, solvent transport, and signal transmission to different but coupled domains [33,34,35,36]. Comparison should identify the instability that each architecture suppresses, the time scale over which stabilization persists, and the transport or response penalty introduced by that intervention.

### 2.4. Hydration-Dependent Network Evolution

The internal structure of a hydrogel fiber continues to evolve after spinning because water uptake and loss change its dimensions, polymer volume fraction, intermolecular interactions, and conductive pathways. Water uptake generally increases the fiber diameter, decreases the polymer volume fraction, and opens pathways for molecular and ionic transport. Moderate hydration can promote ion dissociation, enhance segmental mobility, reduce the elastic modulus, and improve conformal contact with skin or tissue. Excessive swelling, however, may dilute mobile charge carriers, weaken load-bearing junctions, and increase the separation between electronically conductive fillers. Water loss produces an equally non-linear response, particularly in ionically conductive fibers. Partial drying may initially increase the effective ion concentration, whereas continued dehydration restricts ion dissociation and mobility, stiffens the network, and raises the resistance of both the fiber and its contact interfaces. Electronically conductive fibers may respond differently because shrinkage can either shorten the distance between conductive domains or generate cracks and interfacial separation. These changes are not necessarily reversible. Dynamic bonds can exchange, polymer chains can relax, pores can collapse, crystallites can reorganize, and displaced fillers may not recover their original contacts when the initial water content is restored. Repeated hydration and dehydration can produce diameter hysteresis, residual deformation, mechanical softening, and electrical drift even without externally applied strain. Reversible mass uptake alone is consequently insufficient to demonstrate structural recovery; fiber diameter, modulus, conductivity, resistance, and interfacial impedance should also return to their initial values after repeated hydration–dehydration cycles.

Table 2 compares representative fiber architectures according to geometry, hydration regulation, transport behavior, and wearable-use constraints under their reported test conditions. Because these systems differ in composition, charge-transport mechanism, water content, temperature, and deformation protocol, their numerical values should be used to identify architectural trade-offs rather than to establish a direct performance ranking. The nanofiber-templated PPy network achieved an electronic conductivity of approximately 8000 S m^−1^ at a water content of about 65% and a tensile strength of 9.4 MPa, but its elongation was limited to 36%, showing that a stable electronic pathway does not necessarily provide large deformation tolerance [20]. In contrast, the core–shell fiber reached an elongation of approximately 1200% while maintaining an ionic conductivity of about 2 S m^−1^ because the aligned hydrogel core supported axial load transfer and ion transport, while the thin shell slowed solvent loss and improved dimensional stability [25]. The textile-integrated fiber in [37] was distinguished less by its instantaneous gauge factor than by its operation below −80 °C, five-month stability, and retention of function over 1000 deformation cycles, demonstrating the importance of solvent stabilization for long-term signal retention. More generally, porous and hollow fibers shorten radial diffusion distances and facilitate exchange with the surrounding medium, but they are susceptible to rapid dehydration, wall collapse, and dimensional variation. Dense or continuously reinforced structures improve axial continuity and suppress crack localization, although high solid fractions and poorly designed interfaces may slow radial equilibration. Core–shell and gradient architectures offer an intermediate strategy by regulating solvent exchange while preserving a functional core, but stronger environmental isolation generally increases response and recovery times. The operative structure of a hydrogel fiber is initially established by spinning, crosslinking, drawing, and solvent exchange, and subsequently reshaped by hydration throughout service. The next section examines how this evolving hydrated structure governs the axial and radial transport of water, ions, electrons, analytes, and photons.

## 3. Hydration-Coupled Transport in Hydrogel Fibers

Transport in hydrogel fibers is often divided into water diffusion, ionic conduction, electronic conduction, molecular transport, and optical propagation. During operation, however, these processes are coupled through the hydration state of the network. Water controls polymer mobility and pore dimensions, solvates ions and analytes, changes dielectric permittivity and electrode polarization, and alters refractive index and light scattering. A change in humidity, temperature, strain, or sweat exposure can affect several information carriers simultaneously. Transport performance should consequently be interpreted from the hydrated network state in which the measurement is performed rather than treated as a fixed material property.

### 3.1. Water Transport and Dynamic Swelling

Water transport establishes the physical state in which other transport processes occur. Radial diffusion usually dominates equilibration in an isolated solid fiber because of its small diameter, whereas pores, lumens, yarn interstices, and textile junctions can support rapid capillary redistribution along the axial direction. Deformation adds a poroelastic contribution: stretching changes pore volume and solvent chemical potential, compression expels liquid, and cyclic loading can pump water across the fiber surface. During skin contact, sweat absorption competes with evaporation and uptake by surrounding fabrics, producing spatial water gradients that may persist even when the average water content appears stable. The working hydration state is a dynamic balance governed by fiber geometry, environmental boundary conditions, deformation history, and assembly structure.

Existing stabilization strategies address different aspects of this process. Reinforced networks resist irreversible pore collapse while retaining water-accessible transport channels [38,39], whereas hygroscopic cosolvents and soft ionic formulations preserve a mobile solvent phase during drying or cooling [40,41]. Anti-swelling and antifreezing networks instead restrict dimensional change or suppress solvent crystallization [42,43,44]. These mechanisms cannot be evaluated by retained mass alone because a fiber may recover its water content without recovering its diameter, pore structure, modulus, or conductive pathway. Water content, diameter, mechanical response, and resistance should be monitored during the same sorption–desorption cycle. The resulting hysteresis provides a more direct indication of whether water transport produces reversible equilibration or accumulated network damage.

### 3.2. Ionic Transport

Ionic conduction requires ion dissociation, solvation, and migration through a connected liquid phase. Hydration affects both the concentration and mobility of the charge carriers. At high water content, improved solvation and lower viscosity favor ion migration, although excessive swelling may dilute the carriers and lengthen transport pathways. Moderate dehydration can initially raise the effective ion concentration, whereas further water loss promotes ion pairing and coordination with polymer chains, sharply reducing mobility. Temperature, strain, and sweat composition shift these regimes by changing solvent viscosity, network free volume, dissociation equilibrium, and pathway continuity. Deformation also alters fiber length and cross-sectional area while redistributing solvent and mobile ions. The measured resistance contains geometric, concentration, mobility, and contact contributions. At low frequencies, ion accumulation at the electrode interface further produces electrical-double-layer polarization, making capacitance and impedance dependent on frequency and equilibration time.

Figure 4a–c connects ionic transport at the material and device levels. The closed-loop hydroElex platform operated at approximately 0.01–0.1 MΩ at 1 kHz and combined electrophysiological recording with voltage-controlled molecular delivery [45]. A 200 mV stimulus applied for 3 min produced repeatable γ-aminobutyric acid delivery, while seizure suppression in animals required a dosage approximately three orders of magnitude lower than that associated with simulated clinical administration. The corresponding cellulose/bentonite hydrogel reached a compressive strength of 3.2 MPa and a fracture energy of 0.45 MJ m^−3^ while retaining ionic conductivities of 8.99 S m^−1^ at 25 °C and 2.58 S m^−1^ at −20 °C [46]. Its freezing point of −28.9 °C suggests that lithium chloride preserved a mobile aqueous phase, whereas negatively charged galleries between bentonite platelets provided preferential cation-transport pathways. Reinforcement was effective because it stabilized the solvent-filled transport geometry rather than merely increasing strength.

Converting a stabilized ionic pathway into an addressable electrical interface requires spatial control. Localized gold-nanoparticle heating induced phase separation and partial poly(styrene sulfonate) removal in poly(3,4-ethylenedioxythiophene):poly(styrene sulfonate) (PEDOT: PSS), creating connected PEDOT-rich tracks without rigidifying the entire hydrated substrate (Figure 4d,e) [47]. The patterned regions reached 67,000 S m^−1^, retained a lateral resolution of 6 μm in water, and showed an electrode impedance of approximately 6 kΩ at 1 kHz. Material-level ion mobility, patterned electronic continuity, and low-impedance tissue contact must be retained together, while conductivity should be reported with temperature, water content, dimensional change, excitation frequency, and interfacial impedance.

### 3.3. Electronic and Mixed Ionic–Electronic Transport

Electronic transport requires a continuous percolating phase, whereas hydration can separate conductive particles, enlarge tunneling gaps, and reorganize conducting-polymer domains. Spinning partly compensates for these effects by aligning carbon nanotubes, graphene derivatives, MXenes, metal nanowires, liquid metals, or conjugated polymers along the fiber axis. Directionally aligned cellulose and PEDOT:PSS/PVA networks use this axial continuity to maintain long-range conduction [48,49], while anti-swelling matrices limit internal pathway rearrangement [50]. Wet-adhesive silk and intrinsically adhesive PEDOT systems stabilize the external contact instead [51,52]. These strategies act at different structural positions and require different measurements: bulk resistance reflects internal percolation, whereas impedance and adhesion tests reflect the tissue-facing interface. PEDOT-based systems further show that conductivity is governed by chain conformation, phase segregation, solvent content, and processing history rather than nominal polymer loading alone [15].

Mixed ionic–electronic conductors bridge ionic biological signals and electronic circuits by allowing ions to penetrate a hydrated phase and modulate an electronic pathway. This volumetric interaction can increase capacitance and reduce interfacial impedance, but it also introduces coupled relaxation because ion uptake can dope or dedope the conducting phase, induce swelling, and alter mechanical properties. PEDOT:PSS-stabilized liquid-metal particles accommodate deformation through mobile electronic contacts, whereas electrostatic crosslinking incorporates PEDOT:PSS directly into the load-bearing network [53,54]. Dual-conductive epidermal gels add an ion-conducting region at the tissue interface [55], while salting-out and direct ink writing regulate polymer contact and spatial pathway placement [56,57]. The relevant comparison is not the highest initial conductivity, but whether electronic continuity and interfacial impedance remain stable under simultaneous strain and hydration changes.

Figure 5a–d follows pathway preservation from healing to fabrication and external power delivery. A self-healing ionic conductor combined a conductivity of 0.49 ± 0.05 S m^−^^1^ with an adhesion strength of 36.73 ± 2.28 kPa, recovered after damage at temperatures as low as −80 °C, and remained functional for more than 45 d [58]. Electrical recovery, rather than specimen shape alone, is the relevant evidence that dynamic interactions reconstructed the transport path. Liquid-in-liquid printing subsequently produced PEDOT: PSS hydrogel pathways with conductivity up to 301 S m^−1^ at a PEDOT: PSS concentration of 9 mg mL^−1^, while the secondary-network modulus was tuned from approximately 0.1 to 500 kPa [59]. These source-reported values describe a specific printed formulation and measurement protocol; they are not treated here as directly comparable with conductivities measured for other compositions, geometries, temperatures, or hydration states. Transmission of sufficient power through 3 mm of animal skin to operate a near-field-communication chip and a light-emitting diode confirmed continuity across the patterned hydrogel and tissue interface.

The organic/inorganic hybrid extends this coupled continuity to energy storage (Figure 5e,f) [60]. Its porous framework combined ionic transport through the hydrated electrolyte with electronic and redox transport through the solid phase, yielding an areal capacitance of 212.5 mF cm^−2^ and an energy density of 18.89 μWh cm^−2^. More than 81% of the initial capacitance remained after 1000 charge–discharge cycles at 6 mA cm^−2^. The evidence identifies four serially dependent elements: the hydrated conductor, patterned geometry, external interface, and storage or readout unit. Resistance, impedance, calibration slope, and device output should be reassessed after damage, hydration cycling, and deformation rather than inferred from recovery of only one component.

### 3.4. Molecular and Biochemical Transport

Biochemical sensing involves a sequence of hydration-dependent steps. Sweat or interstitial fluid must reach the fiber, target molecules must partition into the hydrated network, analytes must diffuse to recognition or catalytic sites, and reaction products must reach an electrode or optical probe before the sampling region is renewed. A highly swellable network may accelerate initial uptake but retain analytes and slow recovery, whereas a dense network may reject interferents at the cost of reduced sensitivity and longer response time. Quantitative sensing depends on both equilibrium partitioning and transient mass transport rather than on receptor selectivity alone.

A stretchable ionic/electronic bilayer illustrates the value of separating sampling from electrochemical conversion [61]. Its hydrated ionic layer dissolved solid epidermal residues and transported them to the electronic layer, where lactate and cholesterol were detected at limits of 0.51 and 0.26 nmol cm^−2^, respectively. Linear calibration regions had Pearson correlation coefficients of 0.99, and the bilayer reduced motion artifacts by approximately threefold relative to a conventional liquid-sensing interface. The data indicate that dissolution, partitioning, and diffusion determine the analyte concentration presented to the electrode before electrochemical conversion occurs. Bimodal systems, dehydration-resistant gels, mapped arrays, and microneedles can improve retention or spatial access [62,63,64,65], but none removes the effects of evaporation, ionic strength, local flow, or temperature. Quantitative analysis requires controlled renewal of the sample volume or an independent measure of its hydration state.

Biochemical access and environmental stability impose opposing requirements. A permeable and highly hydrated recognition domain facilitates analyte transport but also increases evaporation, swelling, and cross-sensitivity. Antifreezing and self-healing natural-polymer networks retain a mobile solvent phase through hydrogen bonding or dynamic crosslinks [66,67], while printable triple networks define the geometry of the sampling region [68]. Wide-humidity conductors and anti-swelling cellulose networks instead limit changes in solvent content and volume [69,70,71]. Temperature-responsive conductors, core–shell cardiac patches, and carbon-dot tactile gels support a common design principle: sampling, recognition, transport, and mechanical support are easier to calibrate when assigned to spatially distinct domains [72,73,74]. Stabilizing components should be concentrated in the supporting or barrier region rather than uniformly introduced into the recognition phase, where they may impede analyte access [75].

### 3.5. Optical Transport

Hydrogel fibers can guide, scatter, absorb, or emit light, but each process remains sensitive to hydration. Water content and polymer concentration determine the refractive index, while swelling, bending, surface roughness, and internal voids influence propagation loss. Fluorophores and structural colors may additionally respond to polarity, ion coordination, intermolecular spacing, or chemical reaction. Optical readout avoids electrode polarization and provides electrical isolation, but it does not eliminate signal drift caused by changes in fiber geometry or solvent distribution.

In a core–shell alginate/polyacrylamide fiber, formulations containing less than 40 wt% acrylamide exhibited an average optical loss below 0.4 dB cm^−1^ across 400–700 nm [36]. The covalently crosslinked polyacrylamide network supplied elasticity, whereas ionically crosslinked alginate dissipated mechanical energy. The fiber tolerated repeated stretching to 700% strain, and deformation-dependent attenuation enabled distributed sensing over a dynamic strain range of 120%. These results show that a mechanically robust optical pathway can be obtained by coupling persistent and dissipative networks. However, bending, swelling, dye redistribution, and hydration-dependent changes in refractive-index contrast can all modify attenuation. Optical loss should be measured under simultaneous deformation and hydration changes before optical and electrical outputs are treated as independent sensing channels.

### 3.6. Coupling Among Transport Modes

Transport modes are coupled because the same state variables regulate different carriers. Water uptake generally improves ion mobility but may enlarge electronic tunneling gaps and change optical refractive-index contrast. Temperature affects solvent viscosity, ion dissociation, chemical reaction rates, and network modulus. Strain changes fiber geometry, induces poroelastic flow, and reorganizes conductive contacts. In self-powered devices, contact and fluid motion may additionally produce charge separation. A measured output can contain a rapid response to the target stimulus and a slower drift arising from hydration, temperature, or structural relaxation.

At the device level, multiple outputs are meaningful only when each can be assigned to a defined transport or transduction process. Printed conductive hydrogels and bioelectronic patches spatially separate ionic and electronic pathways [76,77,78]. MXene and liquid-metal phases preserve electronic continuity during deformation [79,80,81,82]. Triboelectric generators and amphibious sensors convert contact or fluid motion into charge separation [83,84], whereas flame-resistant, cold-tolerant, and multimodal networks attempt to maintain several pathways within a common operating window [85,86]. Diagnostic systems impose a stricter requirement because an electrical pattern must be traceable to a physiological state [87]. Changes in water content, temperature, and contact area can produce correlated outputs that resemble multimodal sensing even when they arise from the same underlying disturbance. Independent calibration, selective perturbation tests, and response-time analysis are needed to establish whether different outputs represent distinct stimuli.

Table 3 compares the preceding systems by the dominant carrier, the hydration variable controlling its transport, and the expected manifestation of instability in a fiber. Some benchmarks were obtained from bulk hydrogels or related soft devices and are included as mechanistic evidence rather than direct fiber-performance rankings. Across the different transport modes, a common design rule emerges: the active pathway and hydration-control function should either coexist in a sufficiently stable domain or be separated into core, shell, and interfacial regions. Ionic fibers require conductivity measurements across temperature and water activity; electronic fibers require resistance and impedance after swelling and drying; biochemical fibers require sampling kinetics and motion controls; and optical fibers require attenuation measurements under simultaneous bending and hydration changes. These paired measurements are necessary to distinguish a signal generated by the intended stimulus from one caused by evolution of the hydrated fiber itself. The following section considers how these coupled transport processes are converted into measurable outputs.

## 4. Signal Transduction, Fidelity, and Decoupling

A hydrogel fiber becomes useful only when transport is converted into a signal whose physical origin remains identifiable during wear. This requirement is more demanding than obtaining a large response from a freshly prepared specimen because strain, temperature, humidity, sweat composition, and skin contact can simultaneously alter geometry, conductive contacts, ion mobility, solvent distribution, and interfacial impedance. Signal fidelity should be evaluated as a connected sequence: a dominant electrical, electrochemical, optical, or self-powered pathway first converts the target input; hydration and mechanical history perturb that pathway; structural design preserves independent information; and calibration separates the remaining coupling within a defined operating domain.

### 4.1. Electrical, Electrochemical, Optical, and Self-Powered Transduction

In resistive fibers, dimensional changes contribute to the signal, but rearrangement of electronic or ionic pathways usually dominates. The microphase-semiseparated PEDOT: PSS nanofiber/PVA network in [88] reached 300% ultimate strain while maintaining mechanical hysteresis below 1.5%, showing that stretchability need not require large irreversible changes when a continuous electronic phase carries charge and a PVA-rich phase accommodates deformation. Dynamic and nanocomposite networks shift this balance rather than remove it: a PDA-modified CNT/polyacrylamide hydrogel produced a gauge factor of 3.93 at 400% strain, a 76 ms response, and 97.3% self-healing efficiency [89], whereas a PANI/PAA network sustained 2830% elongation and reached a gauge factor of 12.63 over 800–2000% strain but required 222 ms and showed slight baseline drift over 100 cycles [90]. These comparisons make gauge factor meaningful only when the strain interval, response time, cyclic baseline, and mechanical and electrical recovery are reported separately; a high peak sensitivity does not by itself establish a stable sensing state.

Capacitive and iontronic fibers translate changes in electrode spacing, permittivity, or electrical-double-layer area into a signal, while electrochemical fibers additionally depend on analyte flux, reaction kinetics, and interfacial activity. Their small radial dimension can accelerate uptake from sweat, but it also makes output sensitive to sweat rate, evaporation, ion dilution, and changing wet contact area. Optical fibers provide a physically different channel through attenuation, scattering, fluorescence, or resonance, yet swelling still changes refractive index and optical path length. The two-channel EMG/FMG interface reported in Ref. [91] achieved 91.28% gesture-decoding accuracy because the channels supplied complementary information; the result demonstrates system-level identifiability rather than intrinsic material selectivity [91]. Across these mechanisms, controlled flow or humidity, fixed electrode overlap, and limited yarn slippage are necessary if the measured output is to retain a known physical meaning.

Self-powered fibers generate charge or voltage through triboelectric contact electrification, piezoionic redistribution, ionic thermodiffusion, moisture gradients, or hydrovoltaic processes [92,93,94]. Removing an excitation source does not remove environmental dependence, because humidity, contact force, loading rate, and circuit impedance remain part of the transfer function. In the S-PPA hydrogel, a protective surface layer raised tensile strength to 5 MPa, more than eleven times that of the unprotected material, while preserving conductivities of 0.80 and 0.33 S m^−1^ in the inner and outer regions [95]. The result shows that a surface barrier can retard water loss and corrosion without fully blocking transport, but self-powered output must still be measured with hydration and circuit conditions if changes in harvested energy are to be distinguished from changes in material state.

### 4.2. Hydration-Induced Drift, Hysteresis, and Dynamic Response

Hydration drift is a multivariable process rather than a monotonic loss of conductivity. Evaporation reduces the fiber diameter and increases the local concentrations of polymers and ions. Carrier density may rise during the initial stage of water loss, whereas further dehydration densifies the network, restricts ion dissociation and mobility, and increases resistance. Fluid uptake produces the opposite dimensional change but does not necessarily produce the opposite electrical response. Swelling can dilute mobile ions, increase the separation between conductive domains, and alter the effective electrical-double-layer area. Because these effects may offset one another, an apparently stable resistance does not demonstrate that the fiber remains in its original hydrated state. Mass, diameter, impedance, and environmental humidity should be monitored simultaneously, and stability should be evaluated by the retention of calibration under a defined water activity rather than by the absence of visible drying.

Mechanical hysteresis introduces further history dependence through chain relaxation, reversible-bond exchange, interfacial friction, and poroelastic flow. A PEDOT: PSS/PVA sensor combined an ultimate strain of 300% with less than 1.5% hysteresis because its continuous conductive phase maintained electrical continuity while the supporting network accommodated deformation [88]. A PANI/PAA hydrogel reached an elongation of 2830% and recovered 86.2% of its mechanical strength after healing for 24 h, but it dissipated approximately 10% of the applied work and showed a small cyclic baseline shift [90]. A double-dynamic-network conductor further increased the elongation to 3250%, although its resistance changed by 223% at 2000% strain [96]. These results distinguish survivable strain from reversible sensing range and post-deformation baseline recovery. A fiber may remain physically intact at extreme strain while exhibiting substantial resistance drift, so the maximum elongation alone does not define the usable sensing range. For repeated wear, calibration retention and baseline recovery are more relevant than the strain at failure.

Response time must be assigned to the process that limits signal formation. Electronic contact rearrangement can occur within tens of milliseconds, whereas ion polarization, thermal diffusion, and radial analyte transport are generally slower. A carbon-nanotube/polyacrylamide strain sensor responded within 76 ms [89], compared with 222 ms for a polyaniline/poly(acrylic acid) system [90]. A 12.15 μm encapsulated hydrogel temperature sensor instead showed a response time of 0.19 s and a recovery time of 0.08 s over −28 to 95.3 °C, while its elastomer coating limited evaporation [97]. The approximately 10 μm polyurethane nanomesh in Figure 6a–d combined 696% stretchability, 1000 cycles at 100% strain, and a water-vapor transmission rate of 1669.3 g m^−2^ d^−1^, and then supported continuous electrocardiography (ECG) for 8 d with an average signal-to-noise ratio (SNR) of 32.2 ± 1.3 dB [98]. Rapid transport is useful only when permeability, adhesion, mechanical recovery, and hydration stability preserve the calibration and interface over the intended wear period.

### 4.3. Cross-Sensitivity in Multimodal Sensing

Hydration and deformation can alter the same electrical output even when the intended transduction mechanism is defined. The ambiguity increases in multimodal systems because strain changes fiber geometry and ion concentration, temperature affects carrier mobility and reaction kinetics, humidity regulates solvent content, and sweat composition alters conductivity and interfacial potential. An anti-swellable PVA/zwitterionic hydrogel retained an equilibrium swelling ratio of 9% after 30 d in water while maintaining ionic conductivity up to 4.58 S m^−1^, toughness of 518 kJ m^−3^, and a compressive modulus of 8.12 MPa [99]. Restricted water uptake was compatible with ionic transport under the reported conditions. Temperature-responsive devices deliberately use related conductivity or capacitance changes as the target signal [97]. The design objective is not universal environmental insensitivity, but a calibrated mapping between the intended input and output with independent measurement or constraint of the variables that perturb it.

This requirement is illustrated by a 12.15 μm hydrogel temperature sensor that achieved a sensitivity of 24.54% °C^−1^, a resolution of 0.8 °C, and subsecond response and recovery [97]. Its capacitance, however, depends on both permittivity and geometry, allowing mechanical deformation to enter the temperature output unless the active layer is mechanically constrained or paired with an independent strain channel. MXene pressure sensors face a related problem because interlayer water, surface chemistry, microstructure, and electrode geometry jointly influence their resistive and capacitive responses [100]. Multimodal performance should be evaluated using a matrix of simultaneously varied inputs rather than a sequence of isolated single-stimulus tests. Coupling can remain useful when different channels preserve sufficient independent information. A two-channel EMG/FMG interface, for example, achieved a gesture-recognition accuracy of 91.28% by combining complementary electrophysiological and mechanical signals [91]. The relevant criterion is not the complete absence of cross-sensitivity, but whether simultaneous inputs produce reproducible and distinguishable response trajectories across devices, users, hydration levels, and environmental conditions. When different input combinations collapse onto the same trajectory, data processing cannot reconstruct the missing physical information without an additional reference or sensing modality. Structural decoupling is required when calibration alone cannot make the coupled response identifiable.

### 4.4. Structural Decoupling

Structural decoupling assigns different inputs to spatially or physically distinct channels. Janus fibers position functional layers on opposite sides, core–shell fibers isolate a conductive or recognition phase, and multicore fibers carry active and reference channels in parallel. The S-PPA hydrogel demonstrates the underlying principle through a planar configuration: its protective skin increased the tensile strength to 5 MPa while retaining region-dependent conductivity [95]. Applying this design to fibers would allow the outer layer to regulate water exchange, mechanical contact, and interfacial adhesion, while the core maintains the principal signal-transport pathway. Spatial separation alone, however, does not guarantee independent outputs because water, ions, heat, and stress can still transfer radially between adjacent regions. The exchange kinetics and interfacial strain transfer must be quantified under relevant hydration and deformation conditions. An effective reference channel should experience the same temperature, humidity, and mechanical strain as the active channel while remaining insensitive to the target recognition reaction.

Orthogonal transduction provides a complementary means of reducing ambiguity by assigning different stimuli to distinct signal types. Mechanical strain may, for example, be measured resistively, while temperature or hydration is monitored capacitively, electrochemically, or optically. A fish-scale graphene sensor achieved a strain range of up to 82%, gauge factors from 16.2 to 150, a detection limit below 0.1% strain, and stable operation for more than 5000 cycles, providing a robust mechanical sensing channel [101]. These metrics do not establish hydration selectivity because water-induced changes in contact resistance can still resemble deformation-driven rearrangement of the conductive network. Combining the mechanical channel with an independent humidity or impedance measurement could distinguish contact-network evolution from solvent-induced drift. Structural decoupling is effective only when each output is linked to a defined physical variable and its response to non-target stimuli is measured quantitatively.

### 4.5. Computational Decoupling and Calibration

Once the device architecture preserves distinguishable information, computational methods can be used to correct residual coupling. Training datasets should cover hydration level, temperature, strain amplitude, strain rate, and contact pressure, with simultaneous perturbations included in addition to conventional single-factor tests [93]. Features such as baseline resistance, impedance phase angle, relaxation time, and optical intensity can reflect different physical processes more effectively than a single peak amplitude. Combining EMG and FMG channels, for example, produced a gesture-recognition accuracy of 91.28% because the two signals contained complementary physiological and mechanical information [91]. This result should not be assumed to represent performance across independently fabricated devices or different users. Randomly assigning adjacent loading cycles to training and test sets can place nearly identical drift histories in both groups and artificially increase the reported accuracy. As a critical recommendation of the present Review, rather than a validation protocol reported by the authors of Ref. [91], more rigorous evaluation requires leave-device-out and leave-user-out testing under varying environmental conditions. When relevant transport variables can be measured directly, physics-informed compensation is preferable to purely empirical fitting. Temperature and humidity channels can correct changes caused by carrier mobility and evaporation, impedance spectra can indicate hydration state and interface quality, and a reference fiber can capture common-mode drift. Any calibration model should remain restricted to its validated operating range, while predictions beyond the tested water activity, ionic strength, temperature, or strain rate should be reported with explicit uncertainty rather than converted directly into concentration, deformation, or force.

These considerations establish a practical hierarchy for preserving signal fidelity. A dominant transduction pathway must first be established, after which hydration and mechanical history should be constrained or independently measured. Structural or orthogonal channels must then retain sufficient information to distinguish simultaneous inputs, and computational calibration should be applied only within the verified operating domain. Table 4 translates this hierarchy into a diagnostic framework covering hydration drift, liquid swelling, mechanical hysteresis, extreme deformation, thermal cross-sensitivity, contact artifacts, multimodal crosstalk, and device-to-device or user-to-user variation. For each limitation, the table identifies its physical origin, provides representative quantitative evidence, and specifies an appropriate mitigation and validation method. The values should not be used to rank materials because they describe different levels of performance. Swelling ratio, conductivity, modulus, and hysteresis characterize the material or fiber, whereas signal-to-noise ratio, decoding accuracy, and long-term baseline stability depend on the electrodes, interfaces, acquisition hardware, calibration method, and wearing conditions. Maintaining this distinction is necessary when translating an individual hydrogel fiber into a textile and ultimately into an on-body sensing system.

## 5. Device and Textile Integration Across Scales

This section examines whether the hydration-dependent function established in a single fiber is transferred without ambiguity to a yarn, textile, skin interface, circuit connection, and complete wearable system. Each integration step changes both the mechanical transfer function and the boundary conditions for water and solute exchange: twisting and knitting redistribute strain, neighboring filaments create capillary reservoirs, encapsulation changes evaporation and analyte access, and rigid electronics concentrate stress at electrical junctions. The discussion follows a scale sequence from processability and yarn construction to textile architecture, skin contact, circuit interfaces, and system operation. At each level, the relevant question is not whether the preceding component remains intact, but whether the same input still produces a reproducible output after assembly.

### 5.1. Fiber Processability and Yarn Construction

A textile-ready hydrogel fiber requires sufficient continuous length, diameter uniformity, wet strength, bending and knot tolerance, surface durability, and storage stability. Tensile strength and elongation alone do not capture these requirements because winding can impose sustained strain and accelerate dehydration, guides can abrade a soft surface, twisting can expel water or create local stress concentrations, and repeated bending around small radii can disrupt conductive pathways without producing visible fracture. Yarn construction can mitigate some of these limitations. Multifilament twisting distributes defects and introduces redundant conductive pathways, whereas core-spun or wrapped yarns combine a hydrated functional component with a conventional load-bearing sheath. Blending with ordinary textile fibers can further improve handle, porosity, and dimensional stability. These advantages are accompanied by a more complex hydration environment because capillary gaps can retain sweat, covering fibers can slow evaporation, and frictional contacts can create history-dependent resistance. Processability must be evaluated after the fiber has been wound, twisted, stored, and conditioned in the hydration state used during service.

The connection between fabrication and subsequent hydration control is evident in a scalable N-acryloylglycinamide/acrylamide fiber containing 5 wt% LiCl. The fiber reached a tensile strength of 2.27 MPa, an elongation of 900%, and an ionic conductivity of 0.69 S m^−1^ [34]. Heating temporarily converted the physically crosslinked gel into a spinnable viscosol, wet spinning restored the fibrous network, and a poly(methyl acrylate) shell subsequently limited both water loss and uncontrolled water uptake. Processability and service stability were thus provided by different states and components of the same system. Reversible amide associations enabled spinning and self-repair, LiCl maintained mobile charge carriers, and the surface layer regulated solvent exchange. This division of function allowed the fibers to be incorporated into strain-sensing and triboelectric textiles without sacrificing their hydrated conducting state.

### 5.2. Textile Architectures and Device Patterning

Textile architecture determines how macroscopic deformation is transferred to a functional fiber. Woven fabrics provide stable and addressable intersections, knitted structures accommodate large deformation through loop rotation, braids tolerate torsion and radial expansion, and embroidery or localized insertion places sensing elements only where they are required. These structures can either reduce the strain carried by the hydrogel fiber or amplify it through geometrical rearrangement, while also controlling air permeability, moisture transport, pixel density, wiring complexity, and tolerance to local failure. The central question is whether deformation occurs within the fiber, at textile junctions, or through rearrangement of the fabric. A sensor embedded in a knit may respond mainly to loop opening rather than intrinsic fiber extension. This behavior is useful when it is reproducible, but calibration must then be performed at the textile level. Distributed arrays introduce further variables, including contact resistance at crossings, row–column crosstalk, and mechanical coupling between neighboring sensing regions. Glycerol-stabilized ionic fibers woven with conventional cloth, for example, formed a five-channel glove and wristband capable of distinguishing finger and wrist movements [37]. The fibers retained function over 1000 loading cycles, remained usable below −80 °C, and exhibited five months of storage stability. The resulting motion signals nevertheless depended on the fabric architecture, which converted multidirectional movement into different axial strains in individual channels, as well as on the glycerol-containing phase that limited water loss and freezing. These results show why cyclic durability and environmental stability measured for an isolated fiber cannot replace calibration of the assembled textile.

Figure 7a–e relates fiber processing and textile geometry to construct-level deformation, transport, and biological performance. Hofmeister-assisted wet spinning produced gelatin–glycerin fibers that were knitted into an open textile with an elongation of 535.51 ± 38.66% and negligible hysteresis over 10 cycles [102]. Air permeability reached 1107.78 ± 12.30 mm s^−1^ at 100 Pa and 3605.22 ± 8.26 mm s^−1^ at 500 Pa, while the textile remained flexible at −80 °C. In an infected-wound model, the drug-loaded construct achieved more than 70% wound closure by day 7 and 98.85 ± 0.49% by day 14. Loop rotation accommodated macroscopic strain, and interconnected pores maintained vapor and therapeutic transport. The additive-manufacturing routes provide a complementary framework for controlling deposition path and local stimulus response [103], but they are not directly comparable with the knitted textile because their geometries and validation endpoints differ.

### 5.3. Hydrogel Fiber–Skin Interfaces

Skin is rough, curved, hairy, chemically heterogeneous, and continuously moving, so a hydrogel fiber–skin interface must remain conformal while maintaining adhesion and ionic continuity. A sufficiently low modulus allows the interface to follow microscopic surface relief, adhesion limits fluctuations in contact area, and a stable hydrated pathway suppresses impedance changes during sweating and movement. Nanoarchitected wireless EEG electrodes and myelin-sheath-inspired epidermal electrodes address these requirements by separating the soft tissue-contacting phase from a mechanically protective conductive phase [104,105]. This spatial separation is more effective than uniformly increasing crosslink density, which may improve handling but usually reduces conformability. Adhesion must likewise be interpreted together with compliance and removability rather than maximized in isolation. A PVA/borax/PEDOT: PSS epidermal hydrogel combined a compressive modulus of 0.3–3.7 kPa with an adhesion strength of 1.96 N cm^−2^ on pig skin, plastic stretchability above 10,000%, and a gauge factor of 3.88 at 500% strain, while supporting ECG and EMG recording [106]. These values show how a compliant adhesive layer can stabilize contact without requiring a stiff backing. Electrostatic–mechanical adhesion provides an alternative when clean and reversible removal is required [107]. The appropriate design target is a reproducible range of adhesion and interfacial impedance throughout wear rather than the highest attainable peel force.

The nanomesh-reinforced ultrathin hydrogel analyzed quantitatively in Section 4.2 also clarifies the interface-level trade-off [98]. Its open reinforcement preserved vapor transport and limited rupture without fully encapsulating the skin-facing gel. The long recording period cannot, however, be attributed to water retention alone because thickness, adhesion, nanomesh load transfer, wireless-module lifetime, and day-to-day changes in sweat and body temperature contributed simultaneously. The source demonstrated continuous ECG wear for 8 days and reported contact-impedance observations extending to 12 days, but it did not provide cohort-scale evidence sufficient to establish inter-user reproducibility or clinical diagnostic performance. The result is treated as longitudinal human feasibility evidence, not as a clinical trial or proof of a universally stable hydration state.

### 5.4. Hydrogel Fiber–Circuit Interfaces

The junction between a soft ionic or mixed-conducting fiber and an electronic circuit is a frequent failure site because the charge carrier, modulus, and deformation geometry change at the same location. A bicontinuous conducting-polymer hydrogel maintained a conductivity above 1100 S m^−1^, a stretchability exceeding 400%, and a fracture toughness greater than 3300 J m^−2^ in a physiological environment while retaining approximately 80% water [12]. Its continuous PEDOT: PSS-rich phase provided electronic transport, while the hydrated polyurethane phase carried mechanical load. A printed PEDOT: PSS/PVA electrical bioadhesive applied the same division of function at the device interface, reaching a conductivity of 1.2 S m^−1^, an interfacial impedance of 20 Ω, a stretchability of 349%, and a toughness of 109 kJ m^−3^ [108]. These results show that low bulk resistance alone cannot ensure a reliable connection. The junction must preserve a continuous electronic pathway, transfer strain away from the contact, maintain adhesion during hydration changes, and resist delamination under repeated bending or stretching. Because the reported impedance also depends on contact area, electrode geometry, test frequency, and measurement configuration, values obtained under different conditions should not be compared directly. Evaluation of the circuit interface should include cyclic resistance, interfacial impedance over the relevant frequency range, adhesion under controlled hydration, and identification of the location at which electrical or mechanical failure begins.

### 5.5. System Integration

Wireless electronics, power management, analog front ends, packaging, and rigid circuit components introduce mechanical loads and sampling constraints that are absent from isolated-fiber measurements. In a self-powered sweat-monitoring platform, each complete sensing and data-acquisition cycle required 4–6 min before Na^+^ and K^+^ concentrations were transmitted wirelessly [109]. This interval resulted from sweat collection, sensor equilibration, power generation, signal processing, and communication, and cannot be inferred from the intrinsic response time of the hydrogel. A flexible sensing fiber connected directly to a rigid battery or circuit board may also generate motion artifacts when the rigid component moves relative to the skin. System design should consequently begin with the required measurement bandwidth, wear duration, communication interval, and allowable power consumption, after which the permitted delay, deformation, and hydration variation can be allocated to individual components.

Integration changes both the mechanical transfer function and the hydration boundary condition at every scale. A functional fiber must remain spinnable, storable, and connectable; a yarn or textile must convert body movement into a reproducible deformation field; the skin interface must stabilize contact without suppressing vapor transport; and the circuit connection must maintain charge transfer despite large differences in stiffness and carrier type. Failure can emerge at any of these transitions even when the preceding component remains structurally intact. Reliability must be verified after yarn formation, textile assembly, skin attachment, circuit connection, and complete system operation rather than inferred from isolated-fiber performance. The following section examines how these coupled structures degrade under realistic wear.

## 6. Reliability Under Wearable Conditions

Wearable reliability is the retention of useful information under coupled environmental, mechanical, chemical, interfacial, and electronic loading. It cannot be inferred from an isolated humidity test or a large number of identical tensile cycles because wear simultaneously introduces variable temperature and humidity, sweat and skin lipids, multidirectional deformation, friction, intermittent contact, and circuit noise. These perturbations act across molecular bonds, hydrated transport pathways, fiber geometry, textile junctions, skin interfaces, and readout electronics; a mechanically intact fiber may deliver an unusable signal, whereas a material with stable water content may still fail through contact drift or chemical fouling. Reliability should consequently be reported through service-relevant boundary conditions and retained signal quality, with duration or cycle count paired with hydration, deformation mode, recovery criterion, and calibration drift.

Here, operando characterization denotes synchronized measurements on the same fiber, textile, or skin-mounted device while hydration and mechanical boundary conditions are actively varied. A minimum protocol should combine gravimetric or vapor-sorption measurements with optical diameter mapping, mechanical load or digital image correlation, and resistance or impedance spectra during programmed relative-humidity, artificial-sweat, temperature, and deformation cycles. Raman, near-infrared, magnetic-resonance, or fluorescence mapping can be added when spatial water populations or ion distributions are central to the mechanism. All channels should share a common time base so that transport lag, structural relaxation, and signal drift can be separated by their characteristic times. The required output is a state trajectory linking water uptake or loss, geometry, stress, charge transport, and calibration, not independent endpoint measurements obtained from different specimens.

### 6.1. Hydration and Environmental Failure

Drying shrinks and stiffens the network, decreases ionic mobility, and promotes interfacial separation, whereas excessive swelling softens the fiber, dilutes mobile species, changes dimensions, and disrupts electronic contacts. Freezing immobilizes solvent and generates local stress, while elevated temperature accelerates evaporation, bond exchange, and chemical degradation; repeated humidity cycles are particularly severe because they drive the material back and forth through these states. A PEDOT: PSS/PVA fiber formed by capillary casting and five freeze–thaw cycles at −20 °C separated these functions among PVA crystallites, a continuous PEDOT: PSS pathway, and a glycerol-rich mobile-solvent reservoir [110]. The approximately 240 μm fiber combined 13.8 MPa strength with 519.9% elongation and remained operational after one year of ambient storage and more than 1000 strain cycles at −60 °C; its gauge factor increased from 0.5 to 5.4 across 0.01–130% strain. The result is more informative than retained mass alone because it links solvent stabilization to mechanics and calibration. Swelling requires a different strategy: a directionally layered PVA/MXene hydrogel reached 2.87 MPa and, after seven days of swelling, retained its structure and strength while resistance changed by only 6% [111]. Directional freezing and salting-out confined transverse expansion and preserved MXene contacts, although the denser architecture may reduce vapor permeability or slow analyte diffusion. Together, the studies show that environmental stability must be judged by the simultaneous retention of dimensions, mechanics, transport, and sensing response.

Figure 8a–d links solvent retention, fatigue resistance, transduction, and recovery within one nanofibrous hybrid architecture [112]. An elastic scaffold arrested cracks while a hygroscopic ionic matrix retained mobile solvent, raising toughness to 24.4 MJ m^−3^, fracture energy to 16.3 kJ m^−2^, and the fatigue threshold to approximately 2950 J m^−2^, compared with about 69 J m^−2^ for the matrix. No obvious crack growth occurred during 10,000 cycles at 200% strain. The gauge factor reached 66.8 at 630% strain, response time was 165–190 ms, and resistance remained stable over 2500 cycles at 200% strain. At 60% RH and 25 °C, mass and shape were retained for 100 d; mechanical and electrical functions recovered within 1 h after complete drying, and no freezing transition was detected down to −90 °C. After damage, 24 h at 80% RH restored 85% of tensile strength. The evidence supports a coupled mechanism in which a persistent scaffold reconstructs load paths and a mobile hygroscopic phase maintains ionic transport.

### 6.2. Mechanical Fatigue and Textile Damage

Figure 9a–f compares two complementary routes to fatigue resistance. A gelatin methacryloyl (GelMA) filament network redistributed crack-tip stress through filament rotation and sliding [113]. At 75 wt% water, FiberGelMA reached 1.1 ± 0.2 MPa strength, 155 ± 41% strain at break, and a fatigue threshold of 2.3 ± 0.5 kJ m^−2^, with additional testing over 30,000 cycles at 1 Hz and under creep at 40% of ultimate strength. Localized PVA crystallinity instead arrested selected crack paths [114], increasing the fatigue threshold from 15 J m^−2^ in the unreinforced gel to 236 J m^−2^ with a crystalline crack-tip region and 290 J m^−2^ with a crystalline mesh; a fully crystalline network containing 18.9 wt% crystallinity exceeded 1000 J m^−2^. Mobile filament networks favor distributed, potentially cell-permissive deformation, whereas patterned crystallinity provides directional crack arrest at the cost of greater structural heterogeneity.

The same logic clarifies the broader literature without reducing it to a list of compositions. Persistent skeletons and fiber architectures preserve load paths [113,115,116,117]. Integrated energy devices and bioadhesive interfaces protect junctions between dissimilar domains [108,118,119]. Organohydrogels, dynamic adhesives, pH-responsive gels, and microfiber nets regulate solvent loss or time-dependent interfaces [120,121,122,123]. Hybrid double-network sensors further couple strain sensing with triboelectric operation [124]. Because these strategies stabilize different variables, they require different validation: fiber-scale reliability should combine tension, bending, twisting, knotting, and yarn-on-yarn abrasion at controlled hydration, while textile-level tests should track junction resistance, loop migration, residual strain, and signal drift. Tensile strength and one-time toughness remain useful material descriptors, but service life is determined by crack-growth resistance, recovery kinetics, and the survival of conductive and interfacial pathways during assembled use.

### 6.3. Chemical, Biological, and Interfacial Degradation

Sweat is a reactive multicomponent fluid rather than a simple hydration source. Its salts, metabolites, proteins, and variable pH exchange ions with the gel, alter swelling pressure, foul electrochemical or optical sites, deactivate enzymes, and corrode metallic contacts; sebum and skin-care products additionally change adhesion and analyte partitioning, microbial growth modifies local chemistry, and washing combines surfactants, immersion, shear, and temperature excursions. Chemical durability must be demonstrated at the recognition and transduction sites as well as in the load-bearing network. A nonenzymatic glucose sensor based on PtNi nanowires and Ni(OH)_2_ nanosheets produced glucose-oxidation activity 2.0 times that of a Pt hydrogel and 270.6 times that of a Ni hydrogel, while the sensing chip remained stable for more than two months [125]. Coupling this chip with a pH sensor and wireless circuit enabled remote sweat-glucose monitoring, showing that catalyst and structural design can remove enzyme deactivation as the dominant aging route. It does not, however, remove fouling, pH-dependent selectivity, or corrosion at external contacts. Artificial sweat, sebum, washing, and rinsing tests should be followed by calibration slope, selectivity, interface impedance, noise, and recovery measurements rather than conductivity alone.

### 6.4. Signal Degradation at Device and Human Levels

System-level failure appears as baseline drift, loss of signal-to-noise ratio, growing crosstalk, intermittent contact, latency, and failed calibration transfer, and may occur without visible fiber damage. Skin slip, cable movement, contact-pressure changes, and deformation of the electrode–skin interface generate motion artifacts, while differences in roughness, perspiration, anatomy, and wearing position limit transfer between users. The wireless hydrogel EEG platform achieved seven-category sustained-attention classification after establishing a stable material contact [104], whereas the self-powered sweat platform required 4–6 min for each wireless sensing set [109]. These systems expose different rate-limiting processes: electrophysiological reliability depends on contact impedance and motion-artifact rejection, whereas biochemical reliability depends on sampling, equilibration, and analyte transport. Appropriate outcomes include waveform correlation with a reference, signal-to-noise ratio over time, percentage of usable data, latency, recalibration frequency, and failure probability across devices and users. Cycle count becomes meaningful only when strain amplitude, rate, multidirectionality, temperature, hydration, and concurrent signal drift are specified.

### 6.5. Reliability Trade-Offs and Stabilization Strategies

Every stabilization route introduces a transport or integration penalty. Humectants and barriers retain water but may slow response; encapsulation suppresses drying but restricts vapor and analyte transport; stronger adhesion improves contact yet can reduce comfort and complicate removal; toughening increases resistance to crack growth but may raise modulus, viscous dissipation, or hysteresis; amplification improves sensitivity but can magnify noise and drift. A rational design sequence should begin with wear duration, temperature and humidity range, deformation mode, sweat exposure, and required signal bandwidth, then define a permissible hydration window rather than a single nominal equilibrium. Hybrid double networks [124], crystalline or fibrous anti-fatigue architectures [114], and conformable self-healing skins [126] control bulk deformation, crack propagation, and contact stability at different levels. Barriers, solvent modifiers, dynamic bonds, and reference channels should be combined only when their effects on response speed, permeability, comfort, chemical access, and calibration are measured under the same service conditions.

Table 5 organizes the evidence by trigger, observable failure, quantitative benchmark, stabilization logic, and required validation. The values are not a direct ranking because humidity, temperature, strain amplitude, geometry, and measured output differ among studies. Their purpose is to distinguish material survival from retained mechanical, electrical, interfacial, or system function. For example, unchanged mass and shape for 100 days [112] establish solvent stability, whereas eight days of electrophysiological recording [98] additionally test contact and signal retention; likewise, 10,000 cycles at 200% strain [112] are informative because amplitude and crack-growth behavior are specified. The most complete reliability claim pairs duration or cycle count with environmental state, deformation mode, drift, and recovery criteria, thereby making the penalty of each stabilization route visible instead of treating durability as a single material property.

## 7. Smart Wearable and Flexible Electronic Applications

Hydrogel-fiber applications are most clearly compared by following the conversion of an external stimulus into a usable and persistent output. Mechanical, electrical, chemical, or thermal inputs first perturb the hydrated network or its interface with skin and tissue. Ions, electrons, molecules, or photons must then move through axial and radial pathways to reach the transduction region, after which yarn construction, textile routing, encapsulation, electrical connection, and power management determine whether the response remains interpretable during wear. This sequence avoids classifying devices only by polymer composition or conductive filler and instead focuses attention on four linked questions: what produces the signal, which transport pathway carries it, which quantitative result supports the proposed mechanism, and under what environmental or mechanical conditions the response remains reliable. The dominant failure mode changes with the application. Mechanical sensors are limited mainly by baseline drift and hysteresis, bioelectrodes by unstable tissue contact and motion artifacts, biochemical sensors by sample history and reference stability, textile arrays by channel mismatch and placement variation, actuators by trajectory repeatability, and autonomous systems by the balance between harvested, stored, and consumed energy. In each case, the working hydration state affects transport and interfacial contact, but its effect must be evaluated through application-specific measurements rather than treated as a universal performance advantage.

### 7.1. Mechanical Sensing, Electronic Skin, and Motion Monitoring

Mechanical sensing provides the most direct example of hydration-dependent transduction because deformation simultaneously changes fiber geometry, mobile-ion concentration, polymer-chain mobility, and contacts within a conductive network. These changes can produce large resistive or capacitive responses, yet evaporation, perspiration, temperature, and prior loading can alter the same parameters without a change in body motion. High sensitivity is useful only when the deformation-dependent response can be separated from hydration-induced drift. A PEDOT:PSS/PVA fiber containing glycerol illustrates this balance [110]. Glycerol-mediated hydrogen bonding produced a tensile strength of 13.76 MPa and an elongation at break of 519.9%, while the sensor detected 0.01% strain across a reported range of 0.01% to 130%. The fiber remained flexible after one year of ambient storage and survived 1000 cycles at 10% strain and −60 °C. These results indicate that partial replacement of volatile water preserved chain mobility and conductive continuity during drying and freezing. However, retained flexibility after storage does not demonstrate that the original strain calibration was preserved, because mechanical integrity, cyclic repeatability, zero-load resistance, and calibration stability are distinct outcomes. A complementary organohydrogel reached a gauge factor of 8.82, remained usable after 90 days of storage, and achieved a capacitive pressure sensitivity of 9.43 kPa^−^^1^ after surface microstructuring [127]. The comparison shows that the measured response results from both material state and device geometry. Microstructured contact and a percolation-sensitive conductive phase amplify deformation, whereas humectant retention stabilizes the supporting hydrated matrix. These functions should be evaluated together through hysteresis, response and recovery time, baseline drift, and recalibration after humidity and temperature conditioning. A recent PVA/cellulose-nanofibril hydrogel containing silver nanoparticles used hydrogen-bond-mediated network cohesion to support sensitive motion monitoring [17]. Because the reported specimen is a bulk hydrogel sensor rather than a spun fiber or textile, it is included as transferable evidence that hydration-sensitive junctions and conductive-particle spacing must be evaluated together, not as direct proof of fiber-level durability.

The error structure becomes more complex when individual fibers are assembled into electronic skins or textile arrays. Liquid-metal/hydrogel conductors, all-weather graphene gels, and notch-insensitive organohydrogels improve conformal conduction and resistance to local damage [128,129,130], while wireless systems add addressing, transmission, and pattern-classification steps [16,131]. Spatial resolution and recognition accuracy then depend on yarn friction, textile deformation, junction resistance, channel mismatch, and placement reproducibility as strongly as on the gauge factor of one fiber. Adjacent loading cycles also share similar drift histories, so randomly dividing them between training and test datasets can overestimate generalization. Credible motion-monitoring systems should be tested across users, days, garments, and hydration states, with calibration retention assessed after repeated wearing, washing, and environmental conditioning. This requirement also marks the transition to electrophysiology. Mechanical sensors are intentionally designed to make deformation dominate the output, whereas physiological electrodes must transmit pre-existing biological potentials without allowing deformation or hydration changes to become the principal signal.

### 7.2. Physiological Electrophysiology and Soft Bioelectrodes

Electrocardiography, electromyography, electroencephalography, and neural interfaces require low and stable electrode–tissue impedance, reproducible ion-to-electron transfer, conformal contact, and minimal motion artifact. Water supplies a compliant ionic pathway, but drying, sweat accumulation, changes in contact pressure, and cable movement can perturb the same interface through which the physiological potential is measured. Lim et al. separated these functions by combining an ultrathin cellular geometry with a conducting polymer [132]. A 100 μm PAAm cellular hydrogel provided a short diffusion path for oxygen, drugs, and heat, while the addition of 1.8 wt% PEDOT: PSS increased the measured surface area from 2.945 to 11.267 m^2^ g^−1^ and lowered impedance across 1–100 Hz. The evidence assigns different roles to the structural variables: thickness and pore architecture govern mass and heat transfer, whereas PEDOT: PSS content and effective contact area control low-frequency electrical coupling. A densified PEDOT: PSS/PVA double-network hydrogel provides a complementary benchmark [8]. At 5.5 wt% conducting polymer, it reached approximately 1000 S m^−1^ conductivity while retaining about 150% fracture strain, limited swelling, tissue-like softness, and adhesive integration. This combination is more relevant to bioelectrodes than maximum elongation because the interface must accommodate tissue motion without converting that motion into a dominant electrical artifact.

Figure 10a–d consolidates the transport requirements of a multifunctional tissue interface [132]. An ultrathin cellular hydrogel formed the common contact layer, with thickness and pore structure governing oxygen, drug, and heat transport. The same hydrated layer supported transcutaneous oxygen-pressure and tissue-impedance measurements, iontophoresis, and transcutaneous electrical nerve stimulation. Multifunctionality depends on a diffusion layer that remains permeable to molecular exchange and conductive enough for electrical transfer, rather than on simple colocation of several operations. Long-term validation should track impedance spectra, SNR, motion artifact, skin condition, and calibration throughout the wear period under concurrent changes in humidity, pressure, and movement.

Human evidence in this field remains dominated by short feasibility demonstrations or single-platform wear studies rather than powered clinical trials. The 8-day ECG demonstration in Ref. [98] establishes longitudinal wearability under daily-life conditions, whereas the wound-healing study in Figure 7 and the closed-loop seizure study in Ref. [45] are animal experiments and cannot be presented as human clinical validation. Across the literature reviewed here, cohort size, participant diversity, device-to-device variation, comparator agreement, adverse skin events, missing-data fraction, and diagnostic sensitivity or specificity are seldom reported together. Clinical translation requires prospective protocols that predefine the hydration and wear conditions, compare signals with an accepted reference, and report both technical failures and participant-level outcomes. Until such evidence is available, claims should be limited to human feasibility, animal efficacy, or materials performance according to the actual validation level.

### 7.3. Sweat and Biochemical Sensing

Biochemical sensing introduces sample collection and molecular recognition before transduction. Sweat is a continuously changing sample because secretion depends on body site and activity, evaporation concentrates solutes, retained liquid carries information from earlier time points, and skin contamination adds background species. Performance measured in a buffered solution characterizes the recognition element but does not establish wearable accuracy. Gai et al. combined ion-selective PEDOT: PSS membranes, power management, Bluetooth transmission, and a hybrid nanogenerator in a self-powered sweat-analysis platform [109]. The Na^+^ and K^+^ channels exhibited sensitivities of 67.43 and 30.42 mV decade^−1^, respectively, while the nanogenerator produced reported outputs of 15 mA and 60 V at low frequency. These quantities describe separate stages of operation. The slopes quantify concentration-to-voltage conversion, whereas current and voltage describe the available electrical source. Their integration supports wireless ion monitoring, but on-body concentration accuracy still depends on sweat renewal, evaporation, response delay, temperature, and reference-electrode stability. The PtNi(1:3) dual-network hydrogel further shows how architecture affects catalytic recognition [125]. Its glucose-oxidation activity was twice that of a Pt hydrogel and 270.6 times that of a Ni hydrogel, and the nonenzymatic sensor remained functional for more than two months. Interconnected PtNi nanowires provided electronic and catalytic pathways, while Ni(OH)_2_ nanosheets supplied additional active interfaces and structural stabilization. The result supports persistent catalytic activity, but longitudinal physiological accuracy would still require interferent testing, controlled sweat delivery, comparison with an independent assay, and a stable reference channel [133,134,135]. Bioinspired rose-petal microtexturing provides a complementary interface strategy: the textured ion-selective membrane maintained sweat-mediated measurement across a reported 2 mm air gap by improving wettability and liquid recirculation [136]. This result addresses intermittent skin contact, but it does not eliminate the need to quantify sample age, evaporation, and reference-potential drift during motion. Because this membrane was evaluated as a nonfiber sweat interface, it is treated as transferable interface-level evidence rather than direct validation of hydrogel-fiber sensing.

Fiber geometry couples sampling to textile integration because pores, lumens, and yarn interstices can collect sweat radially and move it axially toward the sensing region. The same spaces may retain old sweat, broaden the response, and mix samples collected at different times. Enzymatic systems add oxygen dependence and activity loss, ion-selective systems require a stable reference potential, and optical systems require compensation for bending and water content. These constraints make it preferable in many cases to separate the sampling, recognition, and load-bearing functions rather than permanently embedding every component in one fiber. Replaceable recognition modules can preserve the textile structure while allowing calibration or sensing chemistries to be renewed. End-to-end validation should report concentration accuracy under controlled sweat flux, residence time, evaporation, deformation, and temperature rather than relying only on sensitivity measured under static solution conditions.

### 7.4. Smart Textiles, Distributed Sensing, and Human–Machine Interaction

Textile assembly converts a local sensor into a distributed interface. Knits accommodate large strain, woven crossings form addressable nodes, and embroidery allows localized routing, but these structures introduce yarn friction, pressure-dependent contacts, junction resistance, and placement variability. Before pattern-recognition algorithms are applied, the hydrated material state must be sufficiently reproducible that the model is not learning uncontrolled drift. The antiswelling PVA/MXene hydrogel provides a useful example [111]. Directional freezing formed aligned lamellae, and citrate-induced salting-out increased tensile strength from 17 kPa to 2.87 MPa while retaining 558% fracture strain. During seven days of immersion, the swelling ratio reached 78%, yet strength stabilized near 0.65 MPa and resistivity increased by only 6%. The material also retained repeatable signals over 500 cycles, and the equilibrated swollen state responded faster underwater than the initial unswollen state. The evidence does not show that swelling was eliminated. Instead, orientation and salting-out converted a large but initially evolving volume change into a more reproducible hydrated state that maintained mechanical integrity and conduction.

Figure 11a–e traces underwater stabilization from directional freezing and salting-out to aligned microstructure, crack control, environmental equilibration, and sensing [111]. Mechanical and electrical properties approached a stable state during immersion, after which the material monitored bending and pressure underwater. This sequence supports operation in an equilibrated swollen state but does not establish that a single calibration remains valid indefinitely. Textile-level reliability still requires cross-user, cross-day, and cross-device tests under concurrent deformation and hydration changes, together with explicit measurement of channel mismatch, garment placement, and junction resistance.

### 7.5. Soft Actuation and Robotic Interfaces

Soft robotic fibers may use solvent uptake, ion migration, heat, light, or an electric field to produce motion, but large free deformation alone does not establish useful actuation. The relevant questions are whether the displacement is repeatable under load, whether the conductive pathway survives the local strain field, and whether solvent redistribution changes the trajectory over time. Direct-ink-written hydrogel paths can translate anisotropic network organization into programmed deformation [137], while Janus, helical, and multilayer geometries can confine differential swelling to produce bending, twisting, or contraction. Such architectures must balance rapid solvent transport with dimensional stability. Excessively slow exchange limits response speed, whereas rapid and spatially uncontrolled swelling promotes delamination and resistance drift. Loaded displacement, blocked force, response and recovery time, and cycle-to-cycle trajectory error are consequently more informative than a single free-bending angle.

Hydrogel-assisted microfluidic spinning offers a related interface-control strategy even though the reported fibers function mainly as sensors rather than solvent-driven actuators [138]. A sacrificial alginate-hydrogel sheath enabled continuous processing of silicone oligomer precursors that were otherwise difficult to spin, producing smooth fibers with diameters from 0.04 to 3.70 mm and elongations up to 1300%. Fluidic self-adaptation maintained the core–shell interface during formation, and the resulting 1 m PDMSliq fiber transmitted red, green, and blue light while retaining a reproducible optical response over 1000 cycles at 100% strain. CNT/PDMSliq fibers also supported finger-motion, wrist-pulse, Morse-code, and wireless sensing. This result shows that a temporary hydrated phase can control geometry and interface formation without remaining the final load-bearing material. For robotic interfaces, the broader implication is that processing aids, active layers, and conductive paths should be assigned separate structural roles so that actuation does not destroy sensing continuity.

### 7.6. Energy Harvesting, Storage, and Self-Powered Operation

When sensing, wireless communication, and actuation are integrated, energy becomes a constraint linking material selection, architecture, hydration control, and duty cycle. Hydrogel fibers can function as soft ionic electrodes in triboelectric nanogenerators, while fiber-shaped supercapacitors buffer intermittent charge and supply short power bursts. These components are complementary but must be evaluated differently. A harvester requires voltage, current, and power under a specified force, displacement, and frequency, whereas a storage element requires capacitance, energy and power densities, leakage, and retention under mechanical and environmental cycling. Open-circuit voltage alone does not establish continuous self-powered operation because rectification, storage, sensing, computation, and transmission all consume part of the generated energy [40]. Shuai et al. continuously spun poly(N-acryloyl glycinamide-co-acrylamide) hydrogel fibers and applied a poly(methyl acrylate) sheath to restrict water exchange [34]. The core–shell fiber reached a tensile strength of 2.27 MPa, an elongation of 900%, and a conductivity of 0.69 S m^−^^1^, allowing the yarn to operate as a deformable electrode, strain sensor, and woven triboelectric element. These results show that encapsulation actively stabilizes hydration and the conductive pathway during textile deformation, although output power under a defined mechanical input, rectification loss, storage efficiency, and connected-device operating time are still required to define a practical energy budget. A hybrid triboelectric-magnetoelastic tactile platform further shows how physically distinct self-powered channels can improve multimodal identifiability, reporting material, softness, and roughness recognition accuracies of 99%, 100%, and 95%, respectively [139]. The platform is not a hydrogel-fiber device and is used only as system-level evidence for orthogonal transduction and environmental decoupling. For hydrated textiles, an equivalent claim would additionally require simultaneous measurement of water state and a complete harvested–stored–consumed energy budget.

Jeon et al. addressed the storage side by thermally drawing a tough-hydrogel-based supercapacitor fiber containing PVA hydrogel electrodes and electrolyte, conductive PCL current collectors, and an EVA encapsulation layer [140]. At 1.0 mA cm^−2^, the fiber reached an areal capacitance of 268 mF cm^−2^ and a volumetric capacitance of 18.8 F cm^−3^, with areal energy and power densities of 29.8 µWh cm^−2^ and 8.48 mW cm^−2^. It retained 96.48% of its initial capacitance after 1000 cycles of 90° bending, 99.3% after 2000 charge–discharge cycles, and 92.1% after 20 weeks in phosphate-buffered saline. Capacitance remained above 99% during five weeks of in vivo implantation, and the fibers powered LEDs for peripheral- and central-nervous-system optogenetic stimulation. Thermal treatment increased the areal capacitance of an equivalent coin-cell configuration from 750.8 to 1067 mF cm^−2^, an increase of approximately 42%, because reversible PVA-borate coordination promoted rebonding across the electrode-electrolyte interface and reduced internal resistance. The EVA sheath further limited ion and moisture leakage. These measurements connect electrochemical output to deformation, physiological exposure, interfacial repair, and an actual load, providing stronger evidence of functional storage than capacitance measured only in an undeformed state.

Figure 12a–d compares interface requirements at the sensing and energy-storage ends of a prospective autonomous system. Hydrogel-assisted microfluidic spinning maintained continuous core–shell geometry in optical and resistive fibers during large cyclic deformation [138], whereas a multilayer hydrogel supercapacitor used encapsulation, conductive collection, electrochemical storage, and reversible interfacial bonding to retain output during bending, knotting, prolonged immersion, and implantation [140]. These independently tested systems do not yet constitute a self-powered closed loop. Their comparison instead shows that sensing and storage share a dependence on stable interfaces and controlled solvent exchange. A complete autonomous-system claim would require co-integration and measurement of harvested average power, rectification and storage losses, sensing duty cycle, communication energy, and uninterrupted operating time.

### 7.7. Feedback and Closed-Loop Biointerfaces

Energy integration enables prolonged operation, but closed-loop control requires sensing and response to remain distinguishable. Systems that combine monitoring with stimulation, drug release, actuation, thermal feedback, or display must determine whether a measured change originates from biology or from hydration drift, deformation, temperature, and solvent redistribution caused by the device itself [45,141,142]. Architectural separation can reduce this ambiguity by placing sensing and actuation in different regions, using matched inactive reference channels, or scheduling measurement and stimulation in separate time windows. State estimation can then account for slowly evolving hydration and temperature. Validation should report total loop delay, false-trigger rate, decision-threshold drift, response under simultaneous disturbances, and safe behavior after a channel failure. None of these quantities can be inferred from an isolated gauge factor, ion sensitivity, capacitance, or free-bending test. Table 6 summarizes representative quantitative evidence, the pathway supported by each result, and the additional reliability measurement needed at the application level. The values are not ranked because strain, impedance, ion sensitivity, swelling, and capacitance were measured under different conditions. Taken together, these application-specific validation requirements define the evidence boundary used in Section 8 to distinguish established conclusions from proposed design priorities.

## 8. Conclusions and Perspectives

The broad literature supports three conclusions. First, hydrogel fibers differ from bulk hydrogels because short radial exchange distances coexist with long axial pathways and textile processability. Second, spinning, drawing, cross-sectional compartmentalization, and reinforcement can preserve stress, ionic, electronic, molecular, or optical transport, but each stabilization route changes water and solute exchange. Third, humidity, temperature, sweat, deformation, and interfacial contact can alter several transport and signal variables simultaneously. Working hydration is part of the transduction pathway rather than a secondary storage parameter. These conclusions are supported across multiple material and device classes. By contrast, the proposed hierarchy of axial-pathway preservation, controlled radial exchange, spatial separation, reference channels, and hydration-aware calibration is the integrative design perspective advanced by this Review; it should be tested prospectively rather than treated as an already universal design law.

The main evidence gaps are limited long-term human and in vivo validation, the absence of standardized hydration-conditioning and equilibration criteria, insufficient interlaboratory and inter-device reproducibility, and sparse manufacturing statistics for continuous fibers and textiles. Operando testing should measure mass or water activity, diameter, mechanical state, resistance or impedance, and sensor output on the same specimen and common time base during defined humidity, temperature, artificial-sweat, and deformation cycles. Reports should distinguish ultimate strain from reversible sensing range, initial sensitivity from calibration retention, mass recovery from structural and electrical recovery, and single-fiber performance from assembled-device operation. A minimum standardized dataset should specify conditioning history, equilibration criterion, fiber geometry, test frequency, contact area, cycle amplitude and rate, drift, hysteresis, noise, missing-data fraction, and post-exposure recovery. These requirements would make cross-study comparison possible without implying that heterogeneous source-reported values are directly rankable.

Priority research should connect materials design to scalable and verifiable systems. Continuous spinning, coating, winding, and textile assembly require statistical control of diameter, hydration, junction resistance, and shelf-life stability, together with sustainable solvent, polymer, and end-of-life choices. Multimodal and self-powered devices should use physically independent channels and report complete energy budgets before wireless or closed-loop operation is claimed. Artificial intelligence can correct residual coupling only when training and test sets are separated by device, user, and wearing session; it cannot recover information that the architecture fails to preserve. Hydration-aware digital twins offer a longer-term route to prediction if diffusion, viscoelasticity, charge transport, interface evolution, and uncertainty are parameterized from operando data. Prospective human studies should then compare the textile with accepted reference devices and report comfort, skin response, calibration transfer, and failure probability. These steps define a tractable transition from laboratory demonstrations to reproducible wearable and flexible bioelectronic systems.

## Figures and Tables

**Figure 1:**
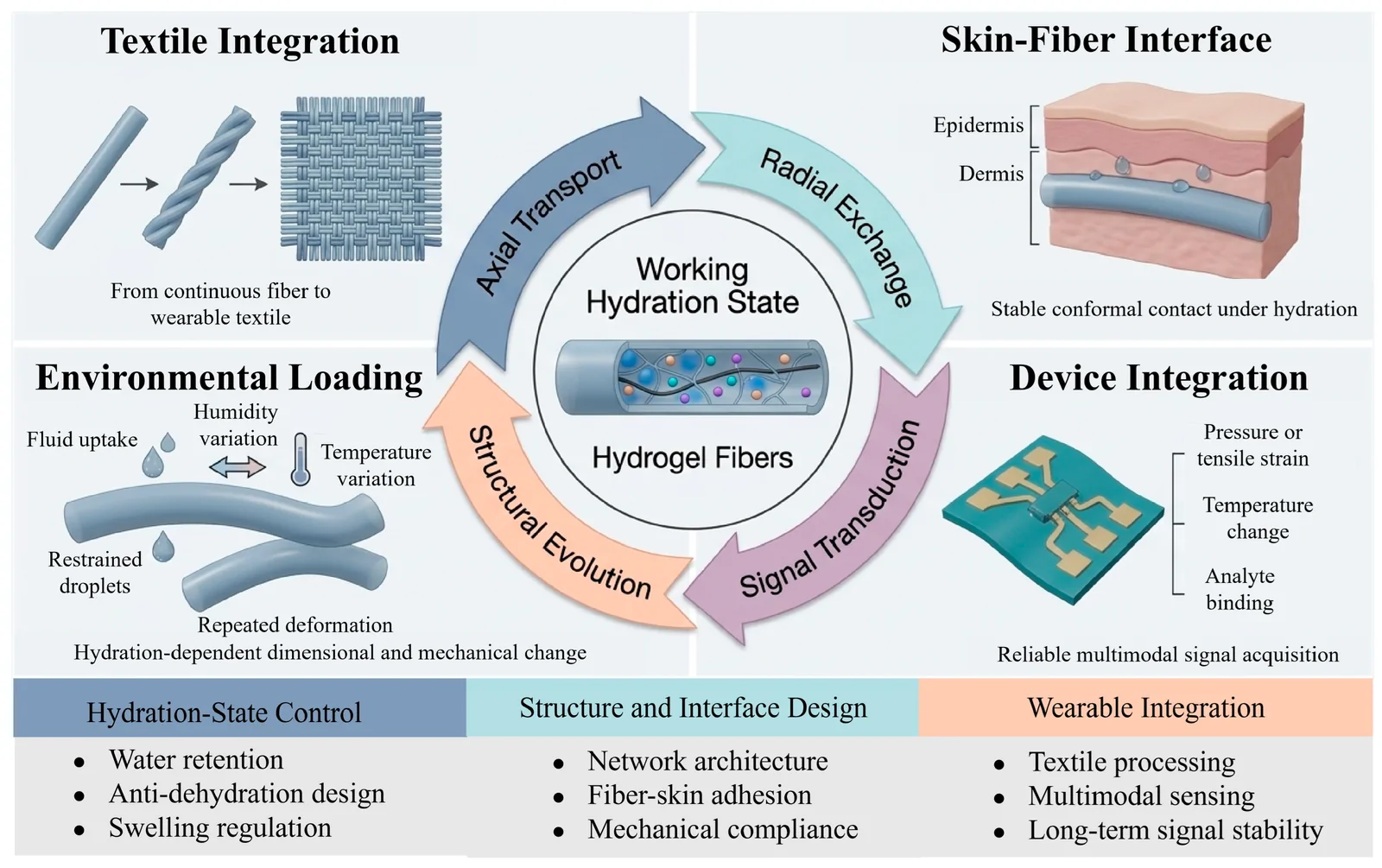
Working-hydration-state framework for hydrogel-fiber bioelectronics. The working hydration state connects environmental exposure with structural evolution, mass and charge transport, signal transduction, and device integration. Water uptake, loss, and redistribution can alter the network structure, dimensions, mechanical properties, interfacial contact, and transport pathways of hydrogel fibers, thereby affecting signal generation and stability. Axial transport, radial exchange, skin-fiber interaction, textile integration, and multimodal signal acquisition are consequently coupled through the hydration state during operation. Reliable wearable performance requires coordinated control of hydration, structure and interface, and device integration to maintain stable signals under environmental and mechanical fluctuations.

**Figure 2:**
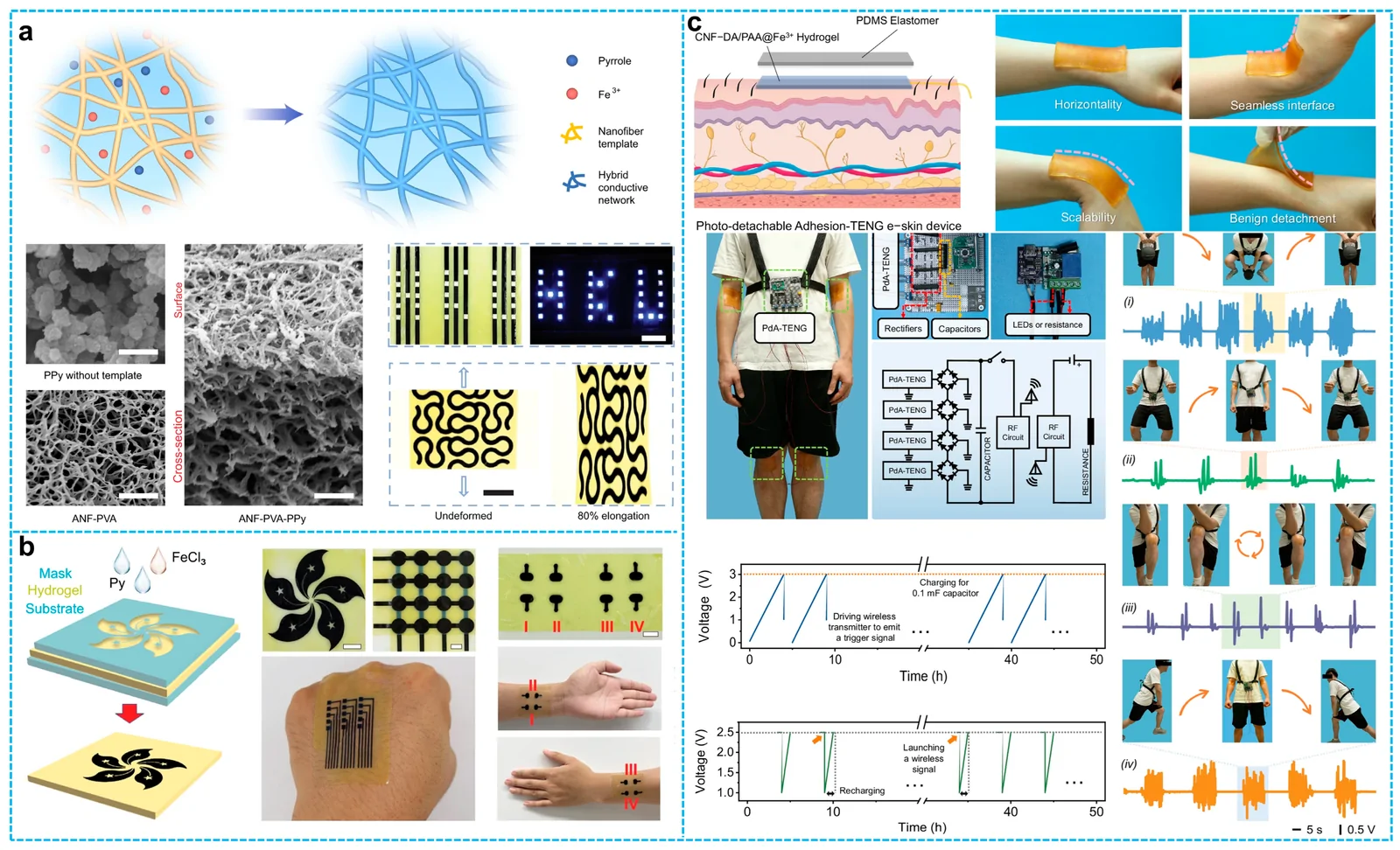
Internal conductive-pathway stabilization and external hydrated-interface regulation. (**a**) Fabrication of an aramid-nanofiber/PVA hydrogel that templates the formation of a percolated polypyrrole network; (**b**) hierarchical structure, electrical performance, and bioelectronic functions of the patterned conductive hydrogel [20]; (**c**) photo-switchable CNF-DA/PAA@Fe^3+^ adhesive hydrogel showing the integration of hydrated ionic conduction, reversible coordination, tissue adhesion, and on-demand detachment in a self-powered electronic-skin system [21].

**Figure 3:**
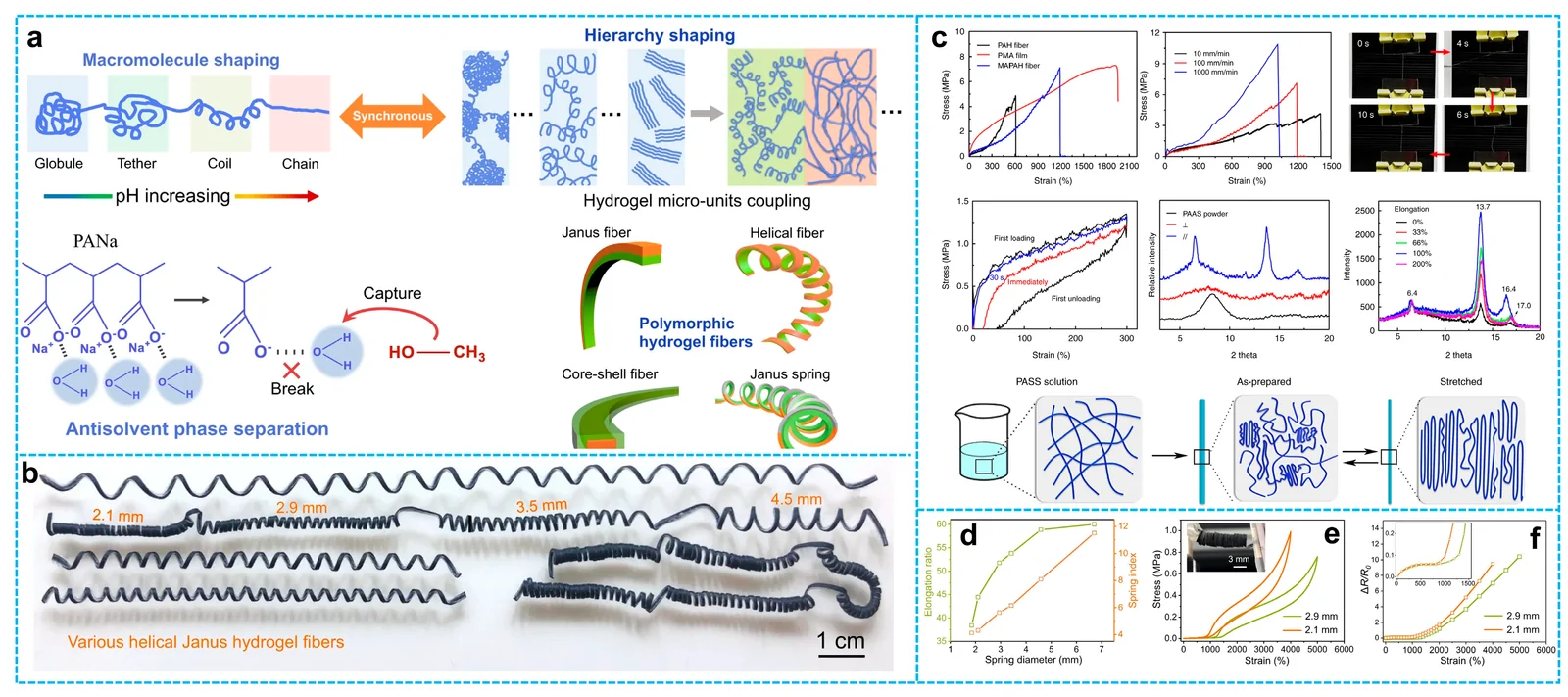
Processing-induced molecular organization, mechanical programming, and hydration protection in hydrogel fibers. (**a**) pH-dependent antisolvent phase separation of sodium polyacrylate and assembly of the resulting microfibers into Janus, core–shell, ribbon, and helical architectures; (**b**) programmed helical structures produced by mismatched deformation of coupled microfiber domains [24]; (**c**) mechanical response, cyclic recovery, X-Ray diffraction, and strain-induced chain alignment of the poly(methyl acrylate)-coated sodium-polyacrylate fiber [25]; (**d**–**f**) relationship among helix diameter, attainable elongation, cyclic loading, and resistance evolution in Janus helical conductors [24].

**Figure 4:**
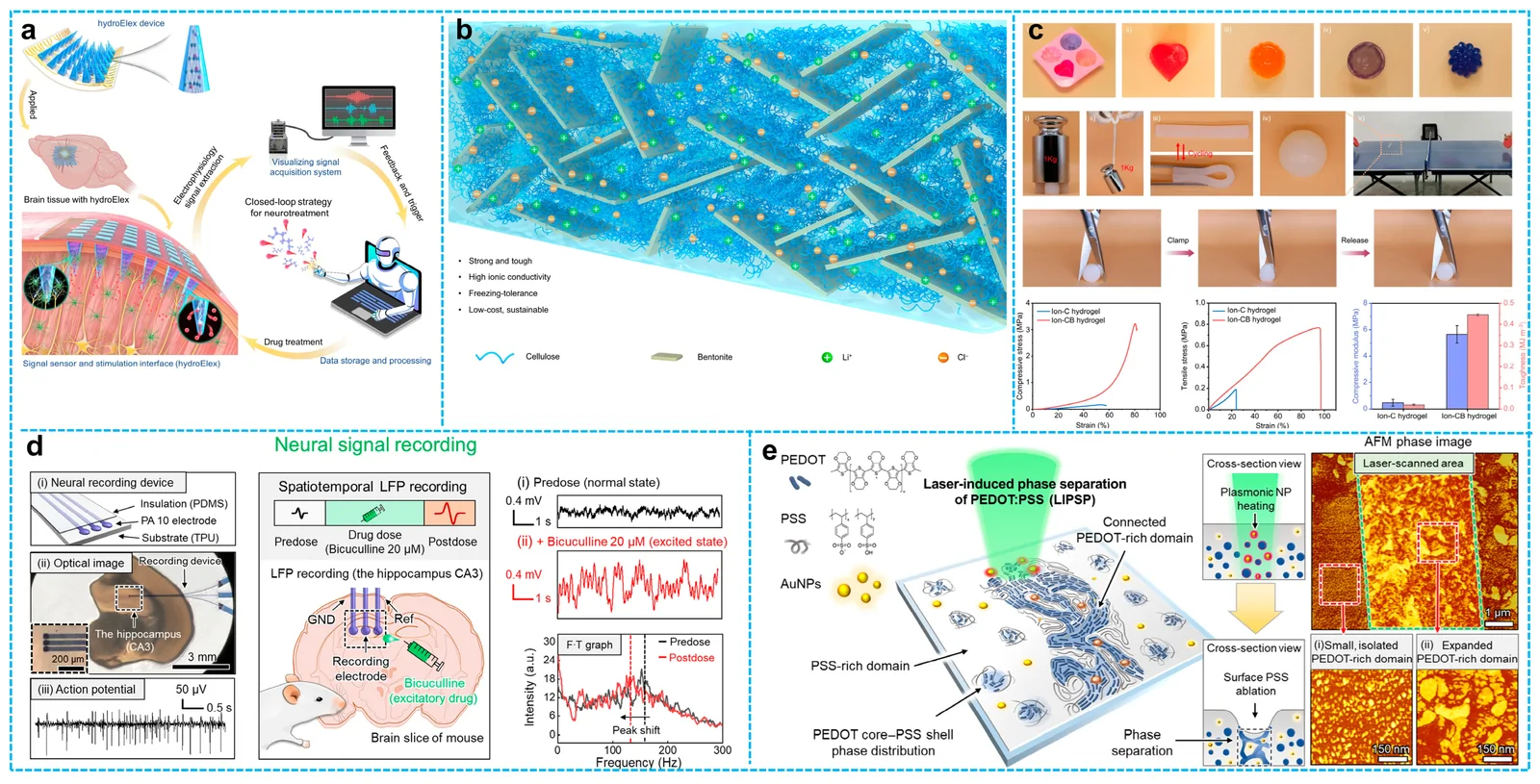
Hydration-stabilized ionic transport from material design to closed-loop bioelectronic operation. (**a**) Closed-loop hydrogel bioelectronics integrating neural recording, seizure recognition, and voltage-triggered antiepileptic drug release [45]; (**b**) mechanical response and (**c**) coordination-reinforced structure of the cellulose/bentonite ionic hydrogel [46]; (**d**) neural recording and stimulation using laser-patterned PEDOT:PSS electrodes and (**e**) formation of connected PEDOT-rich pathways through Au-nanoparticle-mediated heating, phase separation, and partial PSS removal [47].

**Figure 5:**
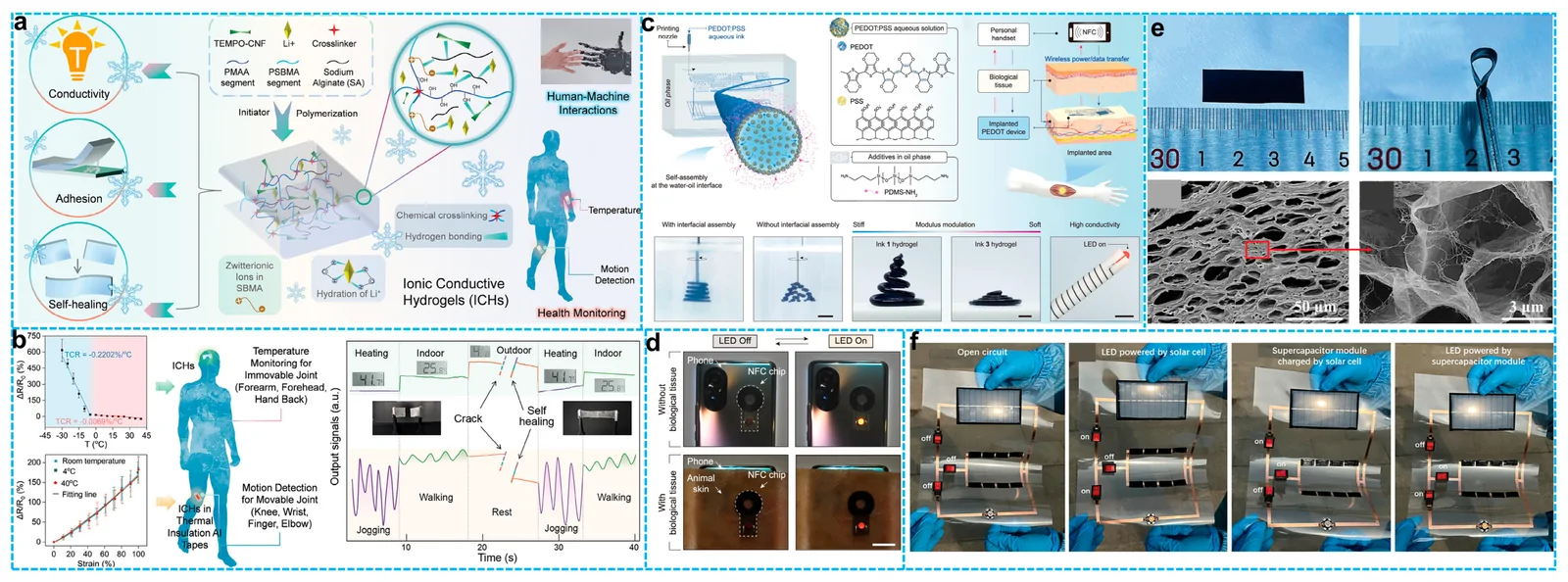
Preservation and integration of mixed ionic–electronic transport in hydrated conductors. (**a**,**b**) Composition, temperature and strain responses, skin monitoring, and signal recovery of a self-healing ionic conductor [58]; (**c**) liquid-in-liquid printing of PEDOT: PSS hydrogels with independently adjustable geometry, modulus, and conductivity and (**d**) wireless power transmission through 3 mm of animal skin to an NFC chip and LED [59]; (**e**) porous organic/inorganic hybrid conductive hydrogel and (**f**) operation of the integrated solar-cell–supercapacitor system during direct power supply, charging, and subsequent LED illumination using stored energy [60].

**Figure 6:**
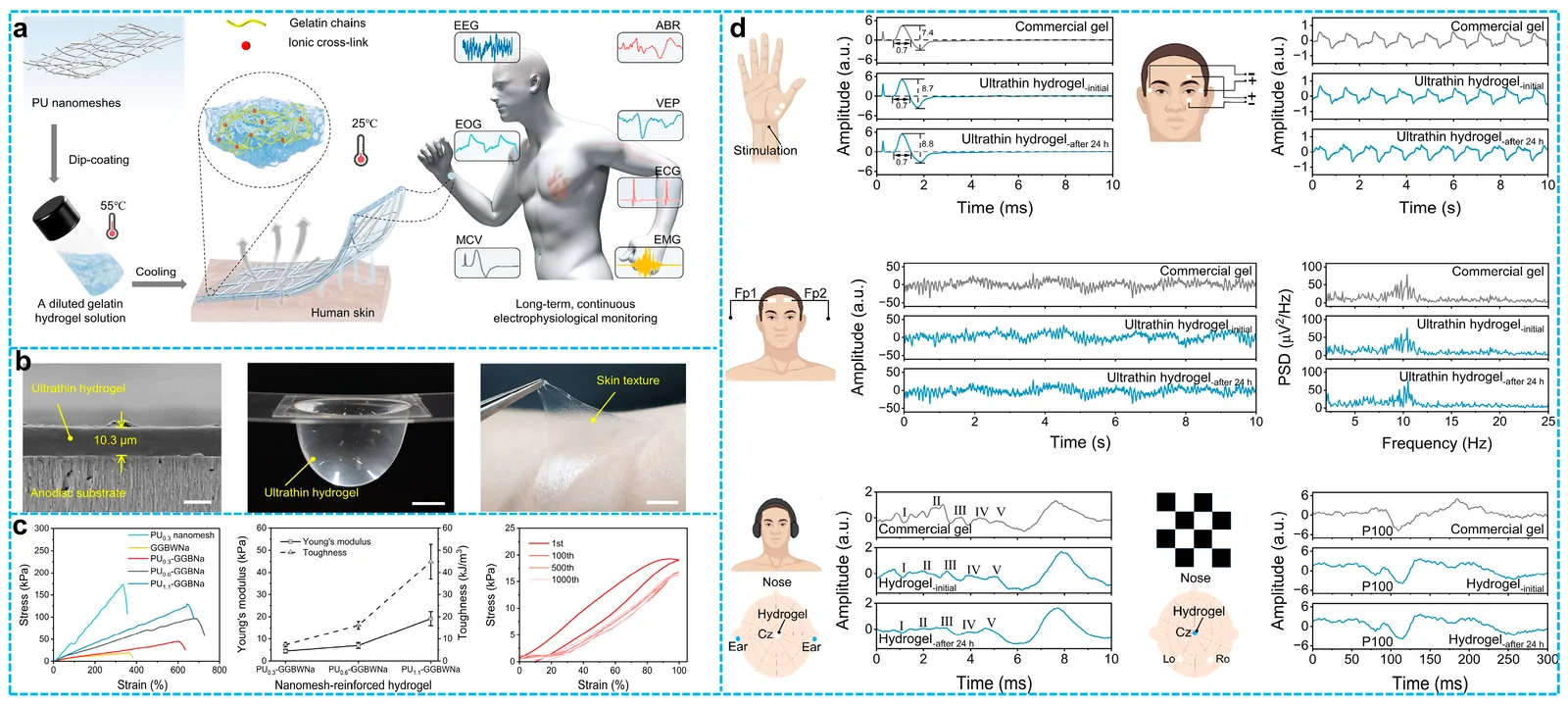
Nanomesh reinforcement couples hydration control to long-term signal fidelity. (**a**) Design and electrophysiological use of a polyurethane (PU) nanomesh-reinforced ultrathin hydrogel. (**b**) The approximately 10 μm hydrogel combines high water loading, skin adhesion, and elasticity. (**c**) Mechanical characterization includes tensile behavior, modulus, toughness, and 1000 cycles at 100% strain. (**d**) Continuous electrophysiological recording demonstrates the device-level consequence of the material design. Reproduced from [98].

**Figure 7:**
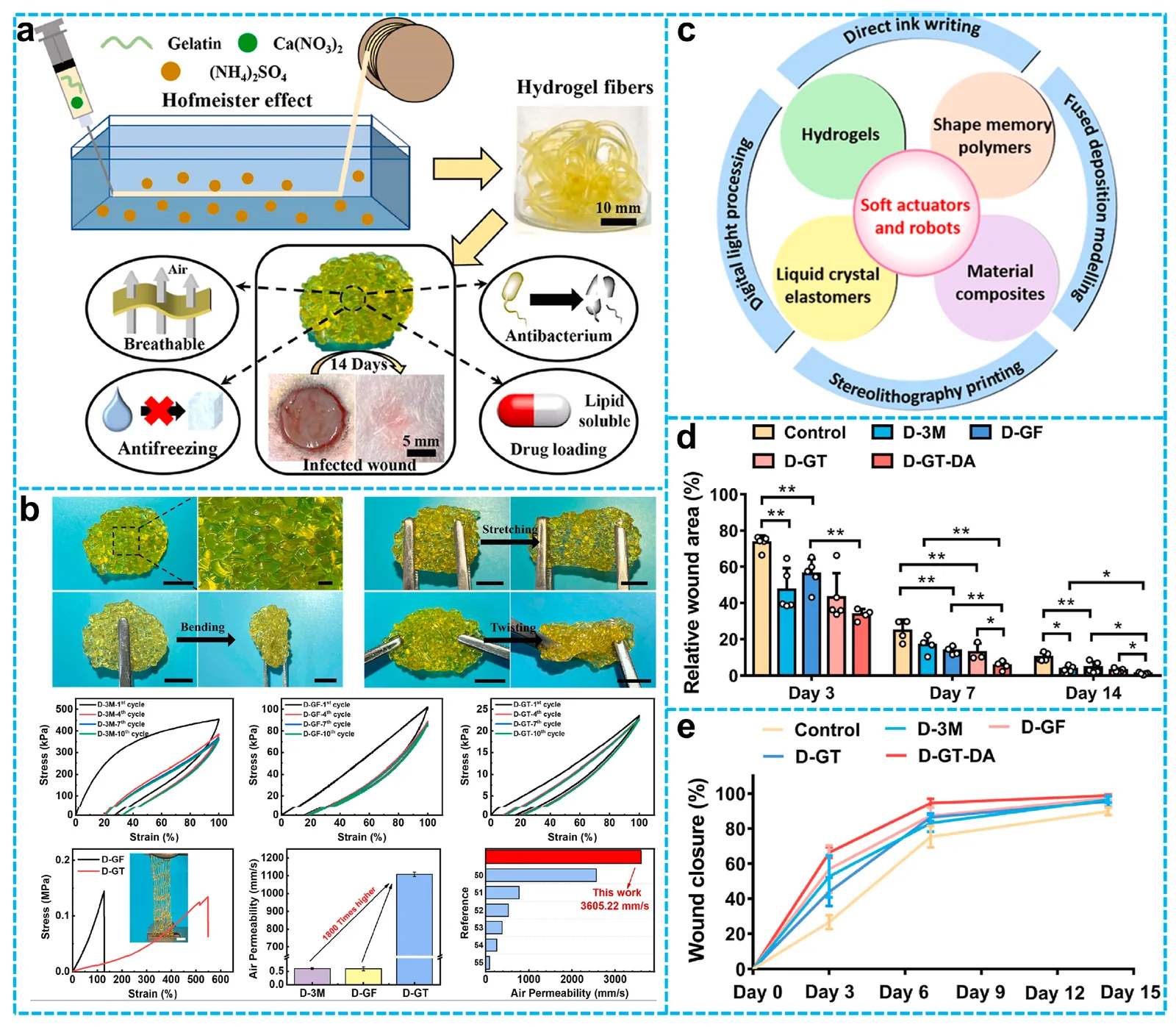
Fiber-to-construct integration. (**a**) Hofmeister-effect-assisted wet spinning and knitting of gelatin-glycerin hydrogel fibers. (**b**) Mechanical deformation and air permeability of the knitted textile and control dressings. (**c**) Additive-manufacturing routes for stimulus-responsive hydrogels and related soft materials. (**d**,**e**) Remaining wound area and healing rate in an infected 1 cm full-thickness dorsal-wound model using 25 male Sprague-Dawley rats allocated to five groups (*n* = 5 per group); wounds were inoculated with 100 µL of Staphylococcus aureus suspension (1 × 10^7^ CFU mL^−1^) and assessed on days 3, 7, and 14. The source reports * *p* < 0.05 and ** *p* < 0.01 but does not specify the statistical test or the definition of the error bars in the figure caption or methods; these omissions preclude a more specific statistical interpretation. Panels (**a**,**b**,**d**,**e**) are adapted from [102]; panel (**c**) is adapted from [103].

**Figure 8:**
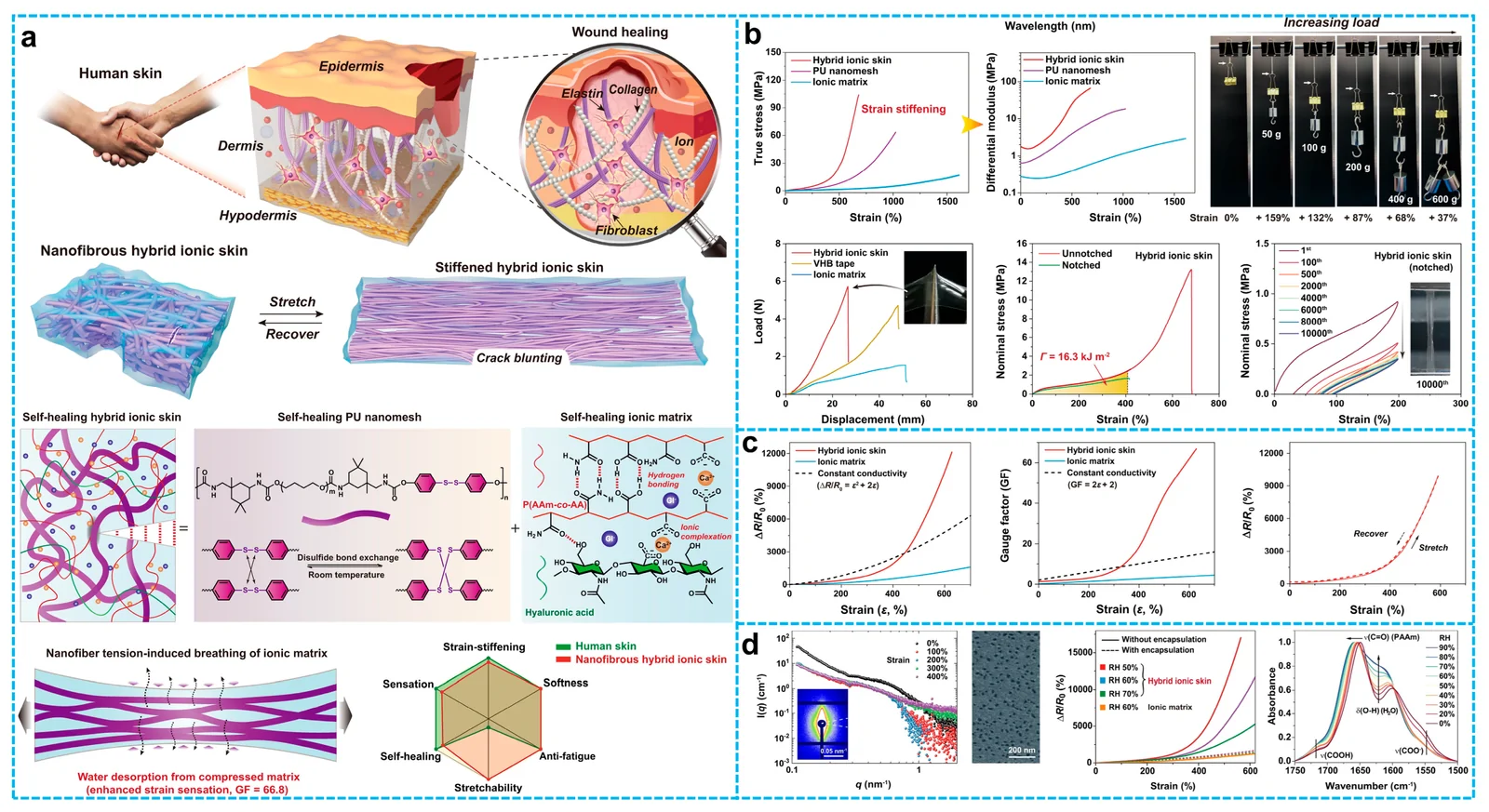
Coupled environmental and fatigue stabilization in a nanofibrous hybrid ionic skin: (**a**) architecture and self-healing; (**b**) fatigue mechanics; (**c**) strain sensing; and (**d**) structural and humidity-dependent electrical analyses [112].

**Figure 9:**
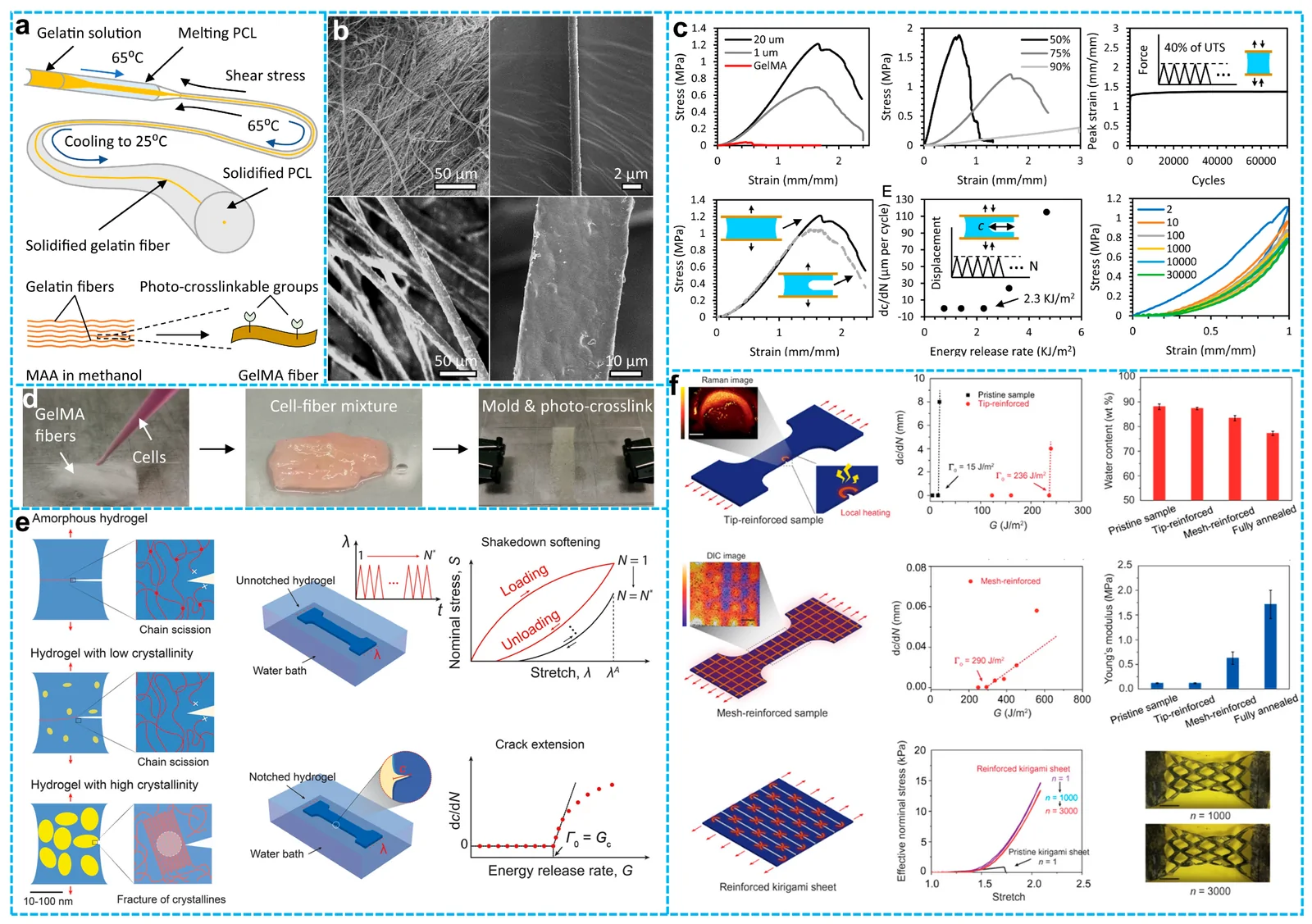
Fiber-scale and molecular routes to fatigue resistance. (**a**) Thermal drawing and chemical functionalization of GelMA fibers. (**b**) SEM images of approximately 1 and 20 μm filaments. (**c**) Water-content- and diameter-dependent tensile, creep, notch, fatigue-threshold, and cyclic tests of FiberGelMA. (**d**) Cell encapsulation by hydration, molding, and photocrosslinking of the fiber network [113]. (**e**) Crack-arrest concept for anti-fatigue hydrogels. (**f**) Crack-tip and mesh-like crystalline reinforcement of PVA, with cyclic kirigami demonstrations [114].

**Figure 10:**
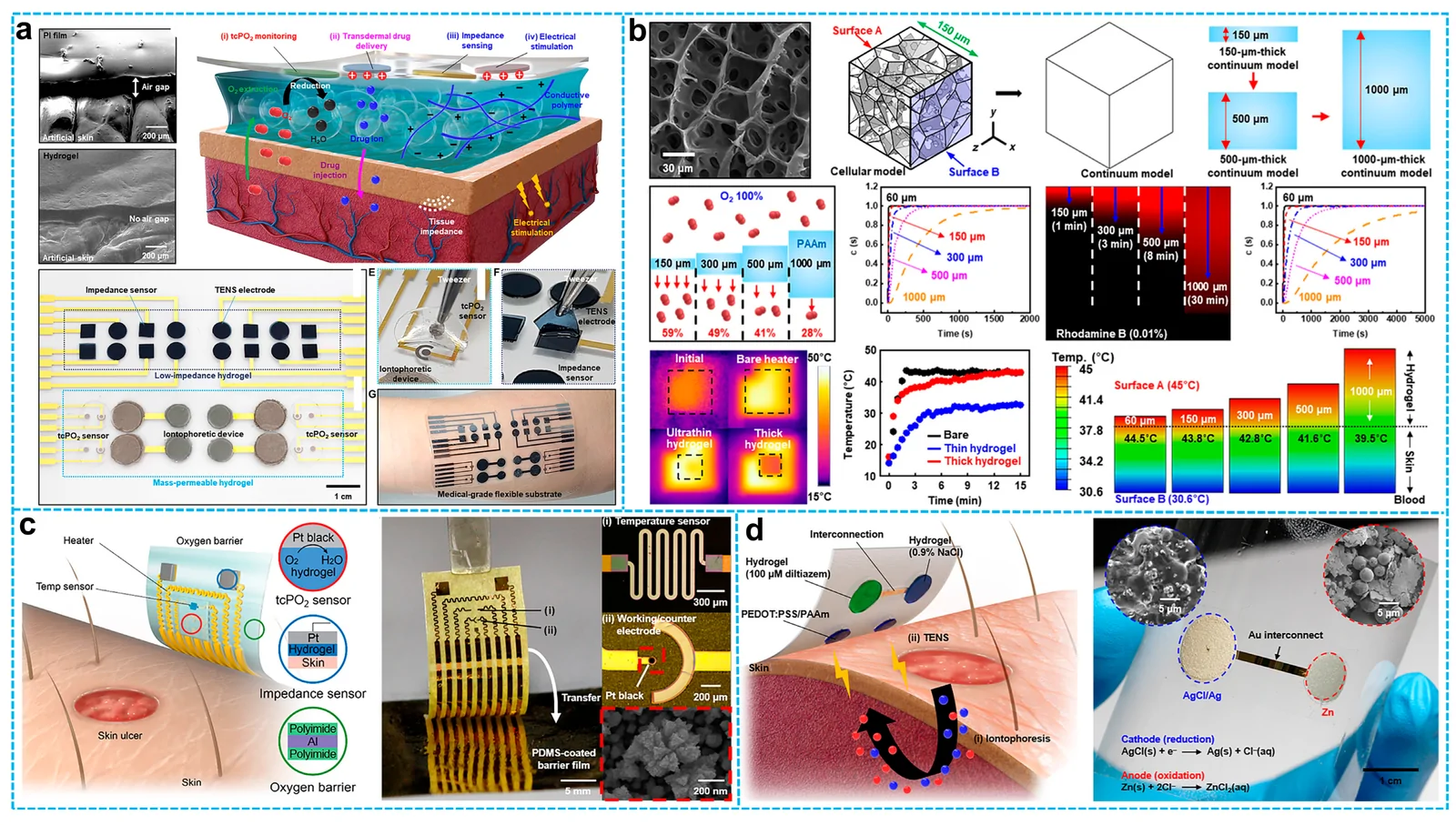
Tissue-like hydrogel interfaces for transcutaneous sensing and stimulation. (**a**) Ultrasoft, permeable, low-impedance contact between skin and a wearable device. (**b**) Thickness-dependent oxygen, drug, and heat transport through the cellular hydrogel. (**c**) Transcutaneous oxygen-pressure and local tissue-impedance measurements. (**d**) Self-powered iontophoresis and transcutaneous electrical nerve stimulation, with AgCl cathode and Zn anode microstructures shown in the insets [132].

**Figure 11:**
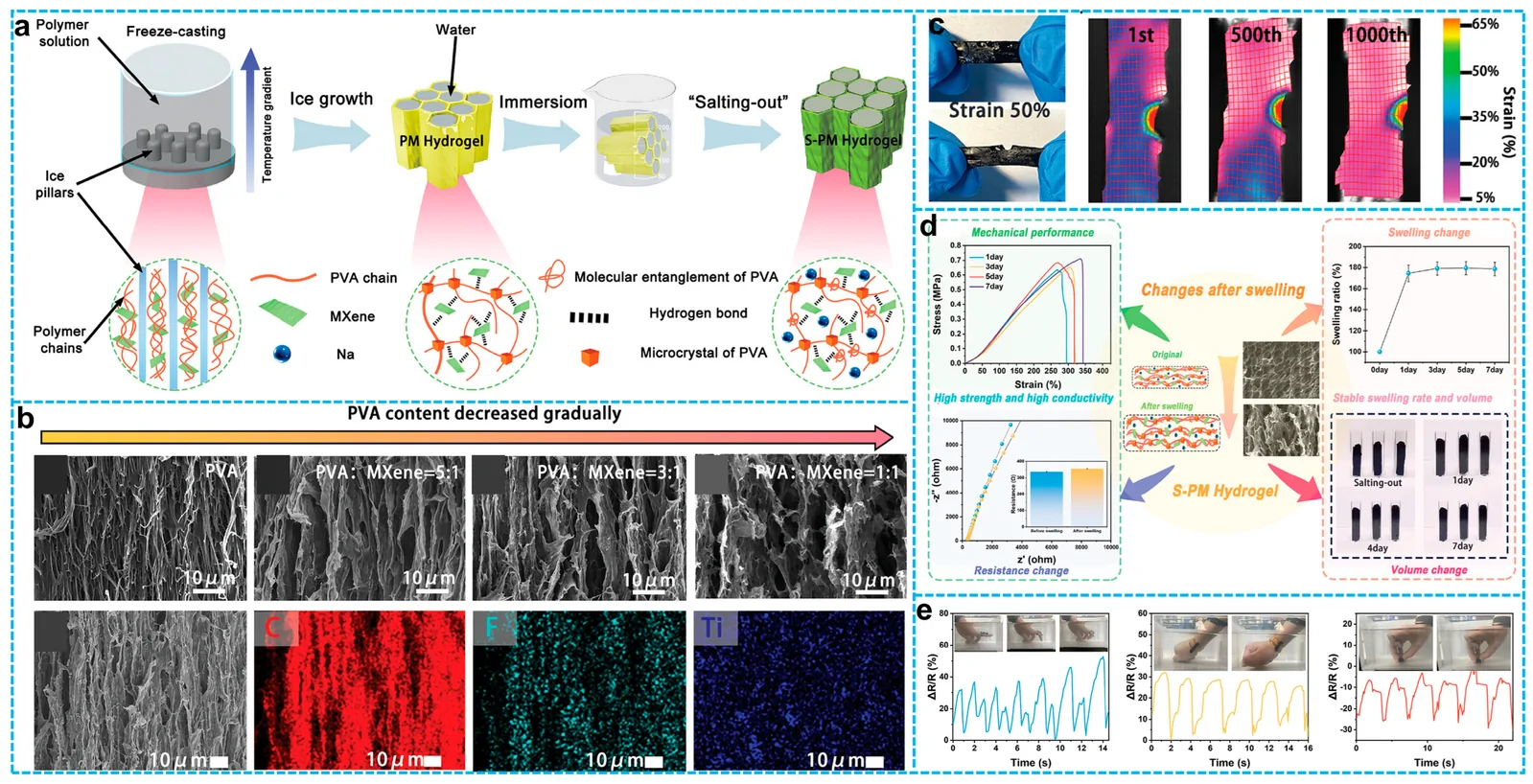
Directional structuring and salting-out of a PVA/MXene hydrogel for underwater sensing. (**a**) Preparation of PM and salt-treated S-PM hydrogels. (**b**) Composition-dependent microstructure and elemental distribution. (**c**) Crack development assessed by imaging and digital image correlation. (**d**) Mechanical, swelling, and electrical evolution during immersion. (**e**) Underwater monitoring of finger and elbow bending and applied pressure [111].

**Figure 12:**
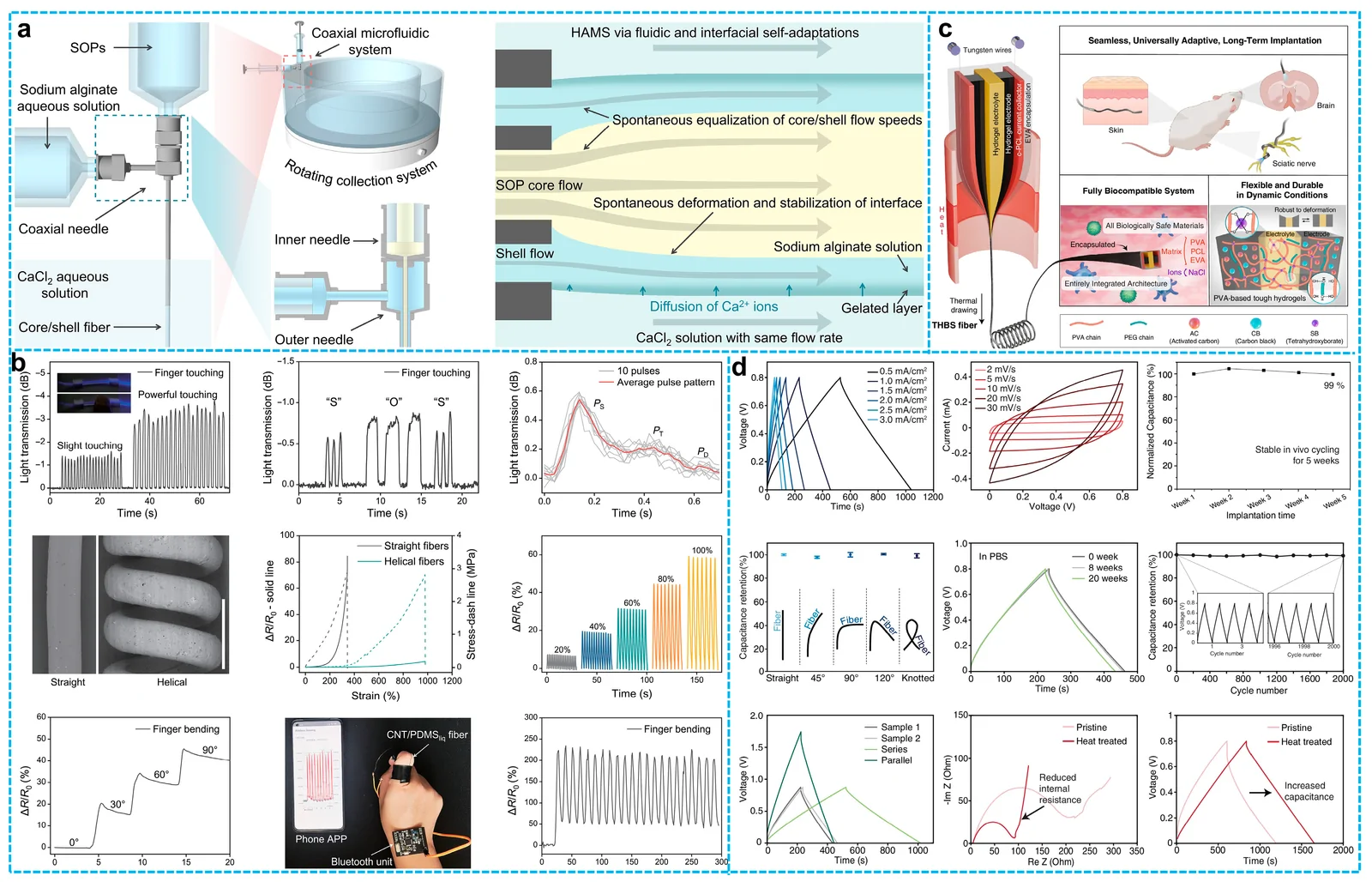
Fiber interfaces for deformable sensing and energy storage. (**a**) Hydrogel-assisted microfluidic formation of silicone-oligomer-precursor/alginate core–shell fibers. (**b**) Optical and resistive responses during motion, pulse, Morse-code, cyclic, and wireless measurements [138]. (**c**) Thermally drawn tough-hydrogel supercapacitor fiber with integrated electrodes, electrolyte, current collectors, encapsulation, and reversible PVA junctions. (**d**) Electrochemical performance during deformation, phosphate-buffered saline immersion, charge–discharge cycling, and thermal interfacial healing [140].

**Table 1:** Comparison of representative reviews with the working-hydration-state framework of the present Review.

Review Scope	Primary Organization	Treatment of Hydration	Contribution Relative to the Present Review	Ref
Hydrogel fibers	Design, synthesis, fabrication, and applications	Hydration is discussed as a material property and processing condition	Does not use the evolving water state as the common variable linking transport, drift, and service reliability	[7]
Conductive hydrogel fibers	Conductive chemistries, spinning, and flexible electronics	Emphasizes conductivity and environmental stability	Limited integration of hydration history with tissue/circuit impedance and calibration retention	[13]
Wearable sensors and soft actuators	Application classes and actuation/sensing functions	Addresses swelling or dehydration within individual examples	Does not provide a cross-application hydration-dependent validation framework	[14]
PEDOT-based conductive hydrogels	Polymer chemistry, processing, and bioelectronic uses	Relates solvent and phase organization to PEDOT transport	Primarily polymer-specific and not centered on fiber-to-textile performance transfer	[15]
Wearable hydrogel temperature sensors	Temperature transduction and device performance	Treats water retention and antifreezing as stabilization strategies	Application-specific; does not unify mechanical, biochemical, optical, and self-powered pathways	[16]
Present Review	Working hydration, coupled transport, signal fidelity, integration, and reliability	Treats spatial water state and exchange kinetics as an operating variable	Distinguishes direct fiber evidence from transferable evidence and links hydration history to retained calibration and service reliability	This review

**Table 2:** Representative hydrogel-fiber architectures and their hydration-dependent transport and performance.

Architecture	Representative System and Geometry	Key Benchmark and Condition	Hydration-Control Mechanism, Advantage, and Limitation	Reference
Nanofiber-reinforced porous network	ANF/PVA framework templating PPy; ≈65 wt% water; percolation near 1 wt% PPy	≈8000 S m^−1^; 9.4 MPa; 36% elongation; ≈20 Ω cm^2^ at 50 Hz	Porosity preserves water access and electronic connectivity, but a higher conductive fraction may reduce deformability.	[20]
Hollow fiber	Ionically crosslinked alginate wall around a cell-accessible lumen	Continuous inner-surface cell seeding; lumen dimensions set by microfluidic processing	The lumen supports axial delivery and the wall radial exchange; collapse and wall thickening increase resistance.	[22]
Reinforced organohydrogel fiber	Continuous reinforcement in an ion-conductive organohydrogel	0.89 S m^−1^ at −40 °C; strain sensing retained during low-temperature deformation	Reinforcement limits crack localization while the solvent phase preserves ion mobility; debonding remains a risk.	[23]
Core–shell fiber	200 ± 20 μm sodium-polyacrylate core; 2–3 μm PMA shell; 25 ± 3 wt% water	5.6 ± 0.6 MPa; 1180 ± 100% elongation; ≈2 S m^−1^; recovery within 30 s after 300% strain	The core carries axial load and ions; the shell slows water loss but may increase equilibration time.	[25]
Janus/helical fiber	Coupled domains with mismatched contraction; helix diameter ≈2.9 mm	Sensing to 1000% strain; ≈30 ms response; 5.7 Hz tracking at 600% strain	Controlled asymmetry converts local contraction into axial extension; uncontrolled hydration gradients cause drift.	[24]
Solvent-stabilized textile fiber	Alginate/PEGDA fiber with glycerol/water and salts	GF 1.04 at 0–50% and 1.87 at 50–200%; <−80 °C; 1000 textile cycles	Solvent stabilization suppresses evaporation and freezing; packing and junction resistance remain device-level constraints.	[37]

**Table 3:** Representative hydration-coupled transport modes and evidence levels in hydrogel fibers and related systems.

Transport Mode, System, and Evidence Level	Key Performance Under Stated Conditions	Hydration-Control Mechanism, Advantage, and Limitation	Ref
Ionic; self-healing LiCl hydrogel; transferable material evidence	0.49 ± 0.05 S m^−1^; 36.73 ± 2.28 kPa adhesion; healing at −80 °C; >45 d function	LiCl retains mobile water and dynamic junctions restore continuity; temperature and evaporation can shift resistance.	[58]
Ionic; cellulose/bentonite hydrogel; transferable material evidence	8.99 S m^−1^ at 25 °C; 2.58 S m^−1^ at −20 °C; freezing point −28.9 °C	Hydrated charged galleries support Li^+^ migration, but mobility remains solvent- and temperature-dependent.	[46]
Electronic; laser-patterned PEDOT: PSS; transferable device evidence	67,000 S m^−1^; 6 μm resolution in water; ≈6 kΩ at 1 kHz	Connected PEDOT-rich tracks preserve electronic conduction; swelling and interface impedance require separate tests.	[47]
Mixed ionic–electronic; printed PEDOT: PSS; transferable device evidence	Up to 301 S m^−1^ at 9 mg mL^−1^; power transfer through 3 mm animal skin	The hydrated matrix enables ionic exchange and PEDOT: PSS carries electrons; interfaces govern device-level loss.	[59]
Energy storage; hybrid hydrogel supercapacitor; transferable system evidence	212.5 mF cm^−2^; 18.89 μWh cm^−2^; >81% after 1000 cycles at 6 mA cm^−2^	Radial ionic access and axial electronic continuity must coexist; drying or delamination interrupts either route.	[60]
Analytes; ionic–electronic bilayer sensor; transferable interface evidence	Detection limits: 0.51 nmol cm^−2^ lactate and 0.26 nmol cm^−2^ cholesterol; ≈3-fold lower motion interference	A permeable layer supplies analytes to the transducer; hydration changes diffusion, partitioning, and apparent concentration.	[61]
Photons; core–shell alginate/PAAm fiber; direct fiber evidence	Optical loss <0.4 dB cm^−1^ at <40 wt% acrylamide; 700% extension; sensing to 120% strain	Hydration sets refractive-index contrast but also changes geometry and attenuation, requiring swelling and bending calibration.	[36]

**Table 4:** Quantitative failure modes, mitigation strategies, and validation requirements for hydration-dependent signal fidelity.

Hydration State and Coupled Failure Mode	Conductivity, Mechanics, and Signal Evidence Under Reported Conditions	Mechanistic Mitigation and Trade-Off	Minimum Validation	Reference
Hydration drift; water exchange changes dimensions, ion concentration, and percolation	≈1% water loss after 21 d; ECG for 8 d with SNR 32.2 ± 1.3 dB	Humectant, permeable encapsulation, or hydration-matched reference	Track mass, diameter, impedance, and output for ≥8 d under humidity and sweat cycling.	[98]
Immersion swelling; uptake changes geometry and carrier concentration	9% equilibrium swelling after 30 d; conductivity to 4.58 S m^−1^	Anti-swelling network and fixed-charge or solvent-activity control	Report swelling, conductivity, modulus, and sensitivity before and after immersion.	[99]
Mechanical hysteresis; viscoelastic and poroelastic recovery is delayed	<1.5% hysteresis to 300% strain; ≈10% dissipation and slight drift over 100 cycles	Elastic load-bearing network, continuous conductor, and rate-aware calibration	Use ≥3 strain rates, dwell and recovery periods, and ≥100 cycles.	[88,90]
Extreme deformation; survival and signal reversibility have different limits	3250% elongation; 223% resistance change at 2000% strain; 86.2% strength recovery after 24 h	Isolated electrodes, recoverable matrix, and dynamic interfaces	Report survivable, reversible, mechanical, electrical, and healing ranges separately.	[90,96]
Thermal crosstalk; temperature changes mobility, permittivity, kinetics, and modulus	12.15 μm sensor: 24.54% °C^−1^; 0.19 s response; 0.08 s recovery; −28 to 95.3 °C	Co-located temperature reference and orthogonal readout	Measure a temperature × strain × humidity matrix with simultaneous inputs.	[97]
Motion/contact artifact; slip and drying alter interface impedance	100 Hz impedance rose from 36.6 kΩ on day 5 to 52.4 kΩ on day 12; ECG remained recordable	Conformal adhesion, ultrathin geometry, soft interconnects, or differential electrodes	Compare sedentary and ambulatory data; track impedance and SNR for ≥1 week.	[98]
Multimodal crosstalk; stimuli share structural and transport pathways	Two-channel EMG/FMG: 91.28% gesture accuracy; separate strain sensor: GF 16.2–150 and >5000 cycles	Janus, core–shell, or multicore design with reference and orthogonal channels	Use independent and simultaneous inputs; report crosstalk and leave-user/device-out accuracy.	[91,101]
Device/user variation; geometry, skin, sweat, and placement differ	Cycle-level random splits can overestimate accuracy when adjacent cycles share drift history	Manufacturing control, hierarchical calibration, and uncertainty bounds	Use leave-device-out and leave-user-out tests across hydration and motion states.	[93]

**Table 5:** Quantitative reliability benchmarks, failure signatures, and stabilization trade-offs for hydrogel-fiber and related wearable systems.

Failure Mode, Trigger, and Consequence	Representative Benchmark and Condition	Stabilization Logic and Penalty	Reference
Dehydration/freezing; low RH or temperature causes shrinkage, stiffening, mobility loss, and impedance drift	Mass and shape retained 100 d at 60% RH and 25 °C; function recovered within 1 h after drying; no freezing to −90 °C	Nanofiber scaffold plus hygroscopic CaCl_2_ matrix preserves load paths and ions; salt may add humidity and thermal crosstalk.	[112]
Over-swelling; sweat or immersion causes softening, filler separation, and calibration drift	PVA/MXene: 2.87 MPa; structure and strength retained after 7 d; resistance changed ≈6%	Directional freezing and salting-out constrain expansion; densification may slow vapor or analyte transport.	[111]
Mechanical fatigue; cyclic loading causes hysteresis, cracks, and irreversible resistance	Fatigue threshold ≈2950 versus ≈69 J m^−2^; no obvious crack growth after 10,000 cycles at 200%; PVA reinforcement: 236–290 J m^−2^	Fibers bridge cracks and crystallinity arrests propagation; reinforcement may increase modulus and process complexity.	[112,114]
Interfacial failure; motion, drying, or rigid leads cause slip and impedance fluctuation	≈10 μm gas-permeable nanomesh supported electrophysiological monitoring for 8 d	Ultrathin open reinforcement balances adhesion and vapor transport; handling robustness remains limiting.	[98]
Chemical/biological degradation; fouling, enzymes, or corrosion causes drift	Dual-structure PtNi glucose-sensing hydrogel retained function for >2 months	Nonenzymatic catalysis avoids enzyme deactivation but still requires selectivity, corrosion, and fouling controls.	[125]
System failure; power or wireless interruption causes missing data and latency	Self-powered sweat platform required 4–6 min for each acquisition-and-transmission cycle	Energy harvesting and multiplexing reduce tethering but add latency, circuitry, and power-management constraints.	[109]

**Table 6:** Quantitative application benchmarks, transport pathways, and reliability requirements for hydrogel-fiber bioelectronics.

Application and Target Signal	Representative Evidence and Condition	Dominant Pathway and Integration	Reliability Metric Required	Reference
Motion/tactile sensing; strain, pressure, pulse, respiration	13.76 MPa; 519.9% fracture strain; 0.01% detection limit; 1000 cycles at −60 °C and 10% strain; GF 8.82; 9.43 kPa^−1^	Deformation changes electronic or ionic conduction in fibers and textile contacts.	Hysteresis, zero-load recovery, and calibration retention under combined humidity, temperature, and motion.	[110,127]
Electrophysiology; ECG, EMG, EEG, neural potentials	PEDOT: PSS fiber: ≈1000 S m^−1^ and ≈150% fracture strain; 100 μm interface increased area from 2.945 to 11.267 m^2^ g^−1^	A conformal electrode transfers ionic tissue potentials to electronic circuitry.	Impedance, SNR, motion artifact, and recording continuity over the complete wear period.	[8,132]
Sweat/biochemical sensing; ions, pH, glucose, lactate, metabolites	Na^+^ and K^+^ sensitivities: 67.43 and 30.42 mV decade^−1^; PtNi sensor retained function >2 months	Sampling and diffusion precede ionic or electrochemical conversion in porous or hollow structures.	Accuracy under controlled sweat flux, evaporation, interferents, deformation, and an independent assay.	[109,125]
Smart textiles/human–machine interfaces; gesture, posture, distributed touch	2.87 MPa; 558% strain; ≈6% resistance change after 7 d immersion; 500 sensing cycles	Woven, knitted, or embroidered networks distribute signals across multiple junctions.	Cross-user, cross-day, and cross-device accuracy under simultaneous hydration and deformation.	[111]
Soft actuation/robotic interfaces; bending, contraction, shape change	Hydrogel-assisted fibers: 0.04–3.70 mm diameter; up to 1300% extension; 1000 optical cycles at 100% strain	Solvent or ion transport couples to anisotropic helical, Janus, or layered geometry.	Loaded displacement, blocked force, response and recovery, and trajectory error over repeated cycles.	[137,138]
Energy storage/closed-loop operation; power and feedback	268 mF cm^−2^; 99.3% after 2000 cycles; 92.1% after 20 weeks in PBS; >99% after 5 weeks in vivo	Ionic storage and encapsulated hydrogel interfaces connect to electronic textile circuits.	Net energy balance, uninterrupted operation, loop delay, false-trigger rate, and fail-safe response.	[140]

## Data Availability

The original contributions presented in this study are included in the article. Further inquiries can be directed to the corresponding authors.
